# WHO Environmental Noise Guidelines for the European Region: A Systematic Review on Environmental Noise and Cardiovascular and Metabolic Effects: A Summary

**DOI:** 10.3390/ijerph15020379

**Published:** 2018-02-22

**Authors:** Elise van Kempen, Maribel Casas, Göran Pershagen, Maria Foraster

**Affiliations:** 1 Dutch National Institute for Public Health and the Environment (RIVM), Centre for Sustainability, Environment and Health, P.O.-Box 1, 3729BA Bilthoven, The Netherlands; 2 Barcelona Institute for Global Health (ISGlobal), 08036 Barcelona, Spain; maribel.casas@isglobal.org (M.C.); mariafp@gmail.com (M.F.); 3 Institute of Environmental Medicine, Karolinska Institute, SE-171 77 Stockholm, Sweden; goran.pershagen@ki.se; 4 Swiss Tropical and Public Health Institute, University of Basel, 4002 Basel, Switzerland

**Keywords:** noise exposure, blood pressure, hypertension, ischaemic heart disease, stroke, diabetes, obesity, meta-analysis

## Abstract

To update the current state of evidence and assess its quality, we conducted a systematic review on the effects of environmental noise exposure on the cardio-metabolic systems as input for the new WHO environmental noise guidelines for the European Region. We identified 600 references relating to studies on effects of noise from road, rail and air traffic, and wind turbines on the cardio-metabolic system, published between January 2000 and August 2015. Only 61 studies, investigating different end points, included information enabling estimation of exposure response relationships. These studies were used for meta-analyses, and assessments of the quality of evidence using the Grading of Recommendations Assessment, Development and Evaluation (GRADE). A majority of the studies concerned traffic noise and hypertension, but most were cross-sectional and suffering from a high risk of bias. The most comprehensive evidence was available for road traffic noise and Ischeamic Heart Diseases (IHD). Combining the results of 7 longitudinal studies revealed a Relative Risk (RR) of 1.08 (95% CI: 1.01–1.15) per 10 dB (L_DEN_) for the association between road traffic noise and the incidence of IHD. We rated the quality of this evidence as high. Only a few studies reported on the association between transportation noise and stroke, diabetes, and/or obesity. The quality of evidence for these associations was rated from moderate to very low, depending on transportation noise source and outcome. For a comprehensive assessment of the impact of noise exposure on the cardiovascular and metabolic system, we need more and better quality evidence, primarily based on longitudinal studies.

## 1. Introduction

### 1.1. Aim

In this paper, we present the main results of a systematic review of the literature dealing with observational studies on the association between environmental noise exposure and the cardiovascular and metabolic systems. The aim was to update some of the existing exposure-response relationships, and to evaluate the overall quality of the evidence. The World Health Organisation (WHO) commissioned this systematic review. Its results form important input for the new environmental noise guidelines for the European Region. The WHO requires that new guidelines should be based on the latest scientific knowledge. The complete review can be found in the report published at the website of RIVM (the Dutch National Institute for Public Health and the Environment) via the following link: http://www.rivm.nl/en/Documents_and_publications/Scientific/Reports/2017/november/Cardiovascular_and_metabolic_effects_of_environmental_noise_Systematic_evidence_review_in_the_framework_of_the_development_of_the_WHO_environmental_noise_guidelines_for_the_European_Region [1].

### 1.2. Background

During the past decades, several national and international organizations have made recommendations for protecting human health from the adverse effects of environmental noise exposure. In the existing guidelines [2,3,4,5], the principal noise source of concern was transportation noise, mainly road and air traffic. The health impact of other noise sources, such as rail traffic and wind turbines, was not addressed in these guidelines. However, with the ongoing extension of railway transport facilities, and the substantial growth of wind energy facilities, the number of studies on the impact of noise from rail traffic noise and on wind turbine noise has increased.

The existing guidelines also contain recommendations that specifically deal with the impact of noise on the cardiovascular system. The most common explanation for the effects of noise on the heart and circulatory system, is stress [2,3]. The cardiovascular effects related to noise exposure may also be the consequence of a decrease in sleep quality, caused by noise exposure during the night, among other additional or interrelated mechanisms. Such reactions may also affect the metabolic system.

The most recent environmental noise guidelines from WHO, date back to 2009, and focus on night-time exposure [3]. Meanwhile, new evidence on the relationship between noise exposure and cardiovascular effects has accumulated. Hypertension and ischaemic heart disease have been the main outcomes of concern in observational studies on the impact of noise on the cardiovascular system. In addition, an increasing amount of studies have recently investigated the impact of noise on *other* cardiovascular end-points such as stroke. Furthermore, hypertension is considered as an important risk factor for other cardiovascular outcomes such as stroke and myocardial infarction. Amongst the newly published studies there were also several studies dealing with the possible effects of noise on the metabolic system, in particular with regard to outcomes such as obesity and type 2 diabetes.

In addition, a number of the newly published studies investigated the combined effects of noise and air pollution. People living close to roads, are exposed not only to traffic noise, but also to air pollution generated by traffic. Previous studies have shown a relationship between air pollution and cardiovascular disease [6,7]. Since air pollution and noise from road traffic share the same source, cardiovascular effects could be attributed to both exposure factors.

The existing environmental noise guidelines also include recommendations that aim to reduce environmental noise exposure in settings where children spend most of their time. However, none of these recommendations takes into account the cardiovascular effects of noise on children. It is possible that people exposed to high levels of noise from an early age, might be at higher risk for cardiovascular problems later in life. Since the publication of the latest environmental noise guidelines in 1999, the number of studies investigating the impact of noise on children’s blood pressure has increased substantially.

## 2. Materials and Methods

### 2.1. Evaluation of Existing Reviews

The first step in this systematic review was to identify and select reviews of “sufficient” quality, that described the impact of exposure to environmental noise from several sources (air, road, rail and wind turbines) on the cardiovascular or metabolic systems, in different settings (at home, at school), and populations (e.g., adults, children).

After an extended search, we identified 37 reviews evaluating available studies into the impact of exposure to environmental noise on the cardiovascular or metabolic systems. By means of the “Measurement tool for the Assessment of Multiple SysTemAtic Reviews” (AMSTAR) [8] we evaluated their quality, and based on the relevance for this whole systematic review, we selected 15 reviews [9,10,11,12,13,14,15,16,17,18,19,20,21,22,23,24,25]. We carried out the evaluation in duplicate (Elise van Kempen and Maria Foraster, and then discussed the results afterwards.

It appeared that most of the studies covered by the selected reviews, reported on the impacts of road and aircraft noise exposure among adults. Nine reviews included one or more meta-analyses, resulting in more than 13 exposure-response relationships. For most available exposure-response relations, the reviewers were not able to provide a quality judgement of the individual studies. For a number of (new) health end-points (e.g., obesity) and/or noise sources (e.g., rail traffic, no reviews or exposure-response relationships were available. 

Following the results of the evaluation of existing reviews, we decided to carry out a new systematic review on the impact of noise on the cardiovascular and metabolic system in order to update some of the existing exposure-response relationships, and to assess the quality of the existing evidence.

### 2.2. Evaluation of Single Studies

#### 2.2.1. Identification and Selection

We identified observational studies on the impact of noise from air, road, and rail traffic and wind turbines on the cardiovascular or metabolic systems published from 2000 until October 2014 in several literature databases (Medline/PubMed, SCOPUS, EMBASE and SCISCEARCH (see Appendix A for the applied search profiles). To ensure that most of the studies could be identified, we manually scanned reports and proceedings in the fields of epidemiology, and noise and health. We supplemented the results of this search with studies that were already identified by means of the 15 reviews, which we evaluated during the first step of this systematic review (see Section 2.1). Overall, we identified more than 600 publications which were screened in duplicate (Elise van Kempen and Maria Foraster) using predefined criteria. We selected 61 studies for data-extraction [26,27,28,29,30,31,32,33,34,35,36,37,38,39,40,41,42,43,44,45,46,47,48,49,50,51,52,53,54,55,56,57,58,59,60,61,62,63,64,65,66,67,68,69,70,71,72,73,74,75,76,77,78,79,80,81,82,83,84,85,86,87,88,89,90,91,92,93,94,95,96,97,98,99,100,101,102,103,104,105,106,107,108,109,110,111,112,113,114,115,116,117,118,119,120,121,122,123,124,125,126,127,128,129,130,131,132,133,134,135], where detailed quantitative information was available on exposure and health outcomes, enabling estimation of exposure-response relationships. However, conducting a systematic review often takes a lot of time. While working on this review, new results became publically available. In order to keep our results more up to date, it was decided to extend our study material with more recent results beyond the studies that had we already identified for the period 2000–October 2014. However, only updated and new results of studies published between November 2014 and August 2015, were included and processed. Consequently, we were able to include the latest results published between November 2014 and August 2015 of several selected studies: DEBATS [26,46], REGICOR [32,33,43,68], SDPP [29,34,73,78,91,106], HUBRO [30,66] and DCH [27,38,51,52,53,63,64,136]. In- and exclusion criteria were extensively described in the complete systematic review [1].

#### 2.2.2. Data Extraction

From the selected 61 studies (described in 113 records), we extracted the following data via a structured data extraction form:

- Data on general study characteristics (e.g., study design, study period, study location);
- Population characteristics (sampling of the study population, number of participants, response- and attrition rate, gender, age;
- Exposure assessment and health outcome assessment, and;
- The results of the study.

We carried out the data extraction in duplicate (Elise van Kempen, Maribel Casas and/or Göran Pershagen) and then discussed the results, with the exception of studies on the impact of wind turbine noise (*n* = 3) and studies on the impact of noise on children’s blood pressure. For those studies data, extraction was carried out by one person only (Elise van Kempen).

In the selected studies we evaluated the risk of bias by means of a checklist developed by the WHO [137]: (i) information bias due to exposure assessment; (ii) bias due to confounding; (iii) bias due to selection of participants; (iv) information bias due to non-objective health outcome assessment, and (v) information bias due to non-blinded health outcome assessment. A protocol of how the studies were scored on each of these five items can be found in Section 3.3 of the complete evidence review available via the link specified in Section 1.1. For each study, the evaluation was carried out independently by two or three reviewers (Elise van Kempen, Maribel Casas and Göran Pershagen). From the scores on the different items, we calculated a total risk of bias score (see also Appendix B for an overview of the risk of bias scores per study).

The main effects under investigation were hypertension, IHD, stroke, type 2 diabetes, change in body mass index (BMI), change in waist circumference, and change in mean blood pressure in children. In order to make a comparison between the studies, we expressed their results in a uniform way and calculated the following outcome variables:

- For studies on the impact of noise on hypertension, IHD, stroke or type 2 diabetes, we calculated the natural logarithm of the Relative Risk (RR) and its variance per 10 dB(A);
- For studies on the impact of noise on children’s blood pressure, we calculated the blood pressure change (mmHg for a noise level increase of 10 dB(A) and its variance for both systolic and diastolic blood pressure; and
- For studies on the impact of noise on obesity markers BMI and waist circumference, we calculated the change in BMI (kg/m^2^) per noise level increase of 10 dB(A) and its variance, and the change in waist circumference (cm) per noise level increase of 10 dB(A) and its variance.

To retain the link with the European Noise Directive (END [138], we expressed noise exposure in L_DEN_. However, most studies did not report an RR per 10 dB (L_DEN_). Where noise exposure was expressed by means of another noise indicator than L_DEN_ (e.g., L_Aeq,16hr_ or L_Aeq,24hr_), a conversion to L_DEN_ was needed. Appendix II of the complete review [1] gives an overview of the conversion rules that we applied.

#### 2.2.3. Data Aggregation

For data-aggregation, we included only estimates from studies that were well matched, adjusted, or stratified for at least age and sex. If more than one risk estimate was available for a study, we used the estimates for men and women separately and for separate age-categories, where possible. After selecting the study estimates, we calculated a pooled estimate using the STATA-command METAN to fit a random-effects model [139]. To test consistency of the effect estimates across studies, we used Cochran’s Q-test [140]. We calculated the I^2^-statistic to reflect the percentage of between-study heterogeneity [141,142]. For some outcomes, we were able to investigate how the summary estimates were affected by sources of heterogeneity. To this end, we carried out a meta-regression analysis using the STATA-command METAREG [141]. Where meta-regression analyses were not possible, we carried out sub-group analyses.

When enough study estimates were available, we attempted to give insight in the extent of publication-bias by means of funnel plots [143]: scatter plots of the studies’ effect estimates (RR per 10 dB) against the inverse of the standard error. Also we applied Egger’s test of publication bias using the STATA-commands METAFUNNEL and METABIAS [144,145].

#### 2.2.4. Assessment of the Quality of Evidence: GRADE

The WHO required us to assess the quality of the evidence that has been retrieved in this review. In other words, we had to assess to what extent we were confident that an estimate of an association between noise and an outcome is likely or unlikely to be changed by further research. 

To this end, we applied a modified version of the GRADE considerations: a systematic and explicit approach to making judgements about quality of evidence [146,147]. In summary, for every outcome, we had to assess the quality of evidence according to several criteria (e.g., study design, study quality, consistency and precision of the results, directness of the evidence, publication bias, whether an exposure-response gradient was present, the magnitude of the effect found, and possible confounding. The scores for the different GRADE criteria are presented in Appendix C to Appendix H as well in Appendices III–VIII of the complete systematic review. How we adapted GRADE for this systematic review is extensively described in Chapter 10 of the complete evidence review [1]. The main divergence from GRADE was that the initial level of certainty was rated “high” for cohort and case-control studies, “low” for cross-sectional studies and “very low” for ecological studies. Furthermore, we upgraded the evidence if the relative risk was 1.5 or higher, but downgraded if based on only one study. GRADE has four levels for the quality of evidence, ranging from “very low” to “high” (see Table 1). The level of the quality of evidence will be linked with the guideline values and recommendations that WHO will include in their environmental noise guidelines.

## 3. Results: Main Findings and Weighing the Quality of the Evidence

In this section, for each outcome the main findings of the review and the conclusions of the weighing of the evidence are presented. The report with the complete findings including the systematic evaluation of the included studies, and the reasoning behind the weighing of the evidence, can be found in the complete systematic review [1].

A note for the reader: since we carried out the literature search for this systematic review, new studies have been published that investigate the associations between transportation noise exposure and metabolic and cardiovascular disease. Unfortunately, owing to time constraints, we were not able to carry out a structured and extensive additional search for new studies published in the period November 2014–March 2017. However, in order to identify at least some of the new studies we were missing, we carried out a search on SCOPUS in March 2017. For this, we applied the same SCOPUS-search profile as was used to identify studies for the current review. In an “ideal” systematic review, we should have included the results of these newly identified studies in the results of the current review, and where necessary updated our results. However, due to time constraints, we have not yet been able to systematically evaluate the newly identified studies. Nevertheless, we have decided to present their results in a narrative way, and attempted to assess how they affect the results of the current review. The differences in results with these recent studies and earlier reviews are described in detail for each outcome in the complete systematic review [1].

### 3.1. Hypertension

We evaluated 40 studies [26,28,30,32,33,35,36,37,40,43,46,49,50,51,55,56,57,60,61,62,63,65,66,67,68,70,73,74,75,76,77,78,80,81,82,83,84,85,86,88,89,90,91,92,94,95,96,97,98,99,101,102,105,106,109,110,112,113,117,118,120,123,126,127,130,131,132,133,134,135,148] that investigated the impact of noise from air, road, and rail traffic and wind turbines on the risk of hypertension. Appendix B presents the separate risk of bias tables. Appendix C presents the different GRADE tables (summarized in Table 2).

There were positive associations between noise from air, road, or rail traffic and hypertension in the cross-sectional studies, which formed the largest part (*n* = 38) of the available evidence (Table 2). After aggregating the results of 26 studies (comprising 154,398 individuals, including 18,957 cases), we derived an RR of 1.05 (95% CI: 1.02–1.08) per 10 dB (L_DEN_) for the association between road traffic noise and the *prevalence* of hypertension. The studies were carried out within the range of approximately 20–80 dB (L_DEN_) [28,30,32,33,35,36,37,43,49,50,55,56,57,61,62,66,67,68,70,75,77,80,82,85,88,89,92,96,97,98,99,109,110,117,118,120,123,126,127,130,131,132,135,149]. For aircraft noise (nine studies), we estimated an RR of 1.05 (95% CI 0.95–1.17) per 10 dB (L_DEN_) (comprising 60,121 residents, including 9487) [28,40,46,50,61,62,74,83,85,94,95,99,102,105,112,113,150]. For rail traffic noise (five studies), we derived an RR of 1.05 (95% CI: 0.88–1.26) per 10 dB (L_DEN_) (comprising of 15,850 individuals, including 2059 cases of hypertension) [28,56,80,82,135]. Although there was evidence for moderate to high heterogeneity among studies, the meta-regression analyses could not reveal clear sources for this observed heterogeneity.

Despite the fact that most studies were able to adjust for important confounders, and were able to ascertain individual exposure levels, we rated the quality of the evidence from the cross-sectional studies mainly as “very low”. This is, among other reasons, because the response rate in many of the studies was lower than 60%. Furthermore, most studies ascertained hypertension by means of self-report only.

In the two evaluated cohort studies that investigated the impact of traffic noise on hypertension, no increased risks were found of hypertension related to traffic noise exposure [51,63,73,78,91,106]. This is confirmed by a recent meta-analysis, including individual data from six cohort studies on the association between road traffic noise and the incidence of hypertension [151]. The reason for this apparent discrepancy in the findings between the cross-sectional and cohort studies is unclear.

Overall, we consider the quality of the evidence *supporting* an association between traffic noise exposure and hypertension as “very low”, indicating that any estimate of effect is very uncertain.

### 3.2. Ischaemic Heart Disease

We evaluated 22 studies [28,42,44,45,47,50,52,53,54,61,62,69,72,75,79,82,83,85,87,90,97,98,99,100,103,107,109,110,111,115,118,120,121,122,123,124,125,128,129,130,131,135] that investigated the association between exposure to noise from air, road, and rail traffic and IHD. Appendix B presents the separate risk of bias tables, and Appendix D presents the different GRADE tables (summarized in Table 3). The majority (*n* = 11) were of cross-sectional design.

The studies that investigated the impact of *air traffic* noise found indications of an increased risk of IHD. Exposure to *aircraft* noise was associated with the *prevalence* of IHD, the *incidence* of IHD, and *mortality* due to IHD [28,42,44,45,47,50,62,69,72,83,85,98,99]. Only the association between *aircraft* noise and the *incidence* of IHD was statistically significant. We estimated an RR of 1.09 (95% CI: 1.04–1.15) per 10 dB (L_DEN_) after aggregating the results of two studies [42,47] comprising of 9,619,082 participants, including 158,977 *incident* cases of IHD. Since most studies on the impact of aircraft noise were of ecological and cross-sectional design (see Table 3), the quality of the evidence from these studies was mostly rated as “very low”. However, the results of the current review are consistent with the results of new longitudinal studies, which reported positive associations between aircraft noise and mortality due to IHD [152,153].

Overall, we rate the quality of the evidence *supporting* an association between *air traffic* noise and IHD as “low”, indicating that further research is very likely to have an important impact on our confidence in the estimate of effect and is likely to change the estimate.

We found evidence that noise from *road traffic* is associated with an increased risk of IHD. An increase in road traffic noise was associated with significant increases in the *prevalence* of IHD, and the *incidence* of IHD. The evidence for a relationship between noise from road traffic and the *incidence* of IHD was the most robust. After combining the results of three cohort studies and four case-control studies [52,53,75,100,107,111,115,118,120,121,122,123,125,130,131] (comprising 67,224 participants, including 7033 *incident* cases of IHD, we found an RR of 1.08 (95% CI: 1.01–1.15) per 10 dB (L_DEN_) for the association between road traffic noise and the *incidence* of IHD within the range of approximately 40–80 dB L_DEN_. This means that if road traffic noise levels increase from 40 to 80 dB (L_DEN_), the RR = 1.36. We rated the quality of the evidence that comes from these studies to be “high”. Supporting evidence came from studies on the association between road traffic noise and the prevalence of IHD. We rated the quality of evidence from these studies as low. The results of the current review are strengthened by the results of several recently published longitudinal studies [152,153].

A visualization of the shape of the association between road traffic noise and the *incidence* of IHD, indicated that the risk of IHD increases continuously for road traffic noise levels from about 50 dB (L_DEN_). This is consistent with the findings of another recent meta-analysis on the association between road traffic noise and IHD [21]. The WHO guidelines of 1999 stated the following: “epidemiological studies show that cardiovascular effects occur after long-term exposure to noise with L_Aeq,24hr_ values of 65–70 dB” [2]. In the WHO Night-noise guidelines, published in 2009, a general threshold of 55 dB (L_Night_) was recommended for protection of cardiovascular disease [3].

Overall, taking into account all available evidence on the association between road traffic noise on IHD, we rate the quality of the evidence *supporting* an association between *road traffic* noise and IHD to be “moderate”, indicating that further research is likely to have an important impact on our confidence in the estimate of effect and may change the estimate. However, for road traffic noise and the incidence of IHD, the quality of the evidence was rated as high.

Compared with noise from road and air traffic, we found only a few studies that investigated the impact of noise from *rail traffic*. These had a cross-sectional design. After aggregating the results of the studies on the association between *rail traffic* noise and the *prevalence* of IHD [28,82,90,135], we found a non-significant RR of 1.18 per 10 dB (L_DEN_).

Overall, we rate the quality of the evidence *supporting* an association between exposure to noise from rail traffic and IHD to be “very low”, indicating that any estimate of effect is very uncertain.

### 3.3. Stroke

Compared with the number of studies on the impact of noise on hypertension and IHD, relatively few studies were available that investigated the impact on stroke (*n* = 9) [27,42,44,45,47,50,52,54,61,62,64,69,72,79,83,85,98,99]. Appendix B presents the separate risk of bias tables, and Appendix E presents the different GRADE tables (summarized in Table 4). 

According to the results of the ecological and cross-sectional studies [28,42,44,45,50,61,62,69,83,85,98,99] an increase in aircraft noise was associated with an increase in the *prevalence* and the *incidence* of stroke. None of these associations was statistically significant (see Table 4). The observations found for the *prevalence* and *incidence* of stroke were supported by the ecological studies [28,42] on the association between air traffic noise and *mortality* due to stroke.

No association between air traffic noise exposure and mortality due to stroke was observed in the evaluated cohort study [72]. This is consistent with the results of new longitudinal studies, which showed no clear indications of an association between aircraft noise and mortality due to stroke [152,153]. 

The results of the studies [27,28,50,52,54,61,62,64,79,83,85,98,99] that investigated the impact of *road* traffic were not consistent. Only for the association between road traffic noise and the *incidence* of stroke, there was a statistically significant RR of 1.14 (95% CI 1.03–1.25) per 10 dB (L_DEN_). This result was based on one cohort study [27,52,64], comprising 51,485 participants, including 1881 incident cases of stroke. 

In the evaluated cross-sectional and ecological studies [27,28,44,45,50,52,54,61,62,64,69,79,83,85,98,99] on the association between road traffic noise and the *prevalence* of stroke or *mortality* due to stroke, no increased risks of stroke due to road traffic noise were observed. This was not consistent with the results of recently published longitudinal studies, which showed that an increase in road traffic noise was statistically significantly associated with an increase in mortality due to stroke [152,153,154]. As part of the current review, only one cross-sectional study [28] was evaluated, which investigated the association between rail traffic noise and the prevalence of stroke. 

Overall, we rate the quality of the evidence *supporting* an association between traffic noise and stroke to be “low”. This indicates that further research is very likely to have an important impact on our confidence in the estimate of effect and is likely to change the estimate.

### 3.4. Diabetes

For the current review, we were able to evaluate seven studies [34,38,60,65,75,76,81,84,86,101] that investigated the association between environmental noise and the risk of diabetes. Four studies [28,34,38,75] investigated the possible impact of transportation (air, road, rail traffic noise. Appendix B presents the separate risk of bias tables, and Appendix F presents the different GRADE tables (summarized in Table 5).

We found two studies [28,34] that investigated the impact of *air* traffic noise on the occurrence of diabetes. In a cross-sectional study [28] on the association between air traffic noise and the *prevalence* of diabetes, a non-significant RR of 1.01 per 10 dB (L_DEN_) was found. In the evaluated cohort study [34] on the association between air traffic noise and the *incidence* of diabetes, no increased risk of diabetes due to air traffic noise was observed (see Table 5).

We found indications that noise from *road traffic* increases the risk of diabetes. The two evaluated cross-sectional studies [28,75] showed an increasing but non-significant trend of the *prevalence* of diabetes with road traffic noise exposure. In the evaluated cohort study [38], an increase in road traffic noise was statistically significantly associated with an increase in the incidence of diabetes. An RR of 1.08 (95% CI: 1.02–1.14) per 10 dB (L_DEN_) across a noise range of approximately 50–70 dB (L_DEN_) was estimated. 

Remarkably, an increase in *rail traffic* noise was associated with a decrease in the risk of diabetes in one cross-sectional study [28] while a cohort study [38] found no statistically significant association. 

Overall, we rate the quality of the evidence *supporting* an association between traffic noise and diabetes to be “low”. This indicates that further research is very likely to have an important impact on our confidence in the estimate of effect and is likely to change the estimate.

### 3.5. Obesity

The number of evaluated studies that investigated the impact of noise on markers of obesity was limited to four [34,136,155,156]: one cohort study and three cross-sectional studies. Appendix B presents the separate risk of bias tables, and Appendix G presents the different GRADE tables (summarized in Table 6). All the studies showed that an increase in traffic noise was associated with an increase in obesity markers, although, according to one study, this was present only in certain subgroups. In the cohort study [34], an increase in aircraft noise of 10 dB (L_DEN_) was associated with a significant increase in waist circumference of 3.46 (95% CI: 2.13–4.77) cm during 8 to 10 years of follow-up (see Table 6). The evidence of traffic noise affecting obesity markers is strengthened by the results of two recent longitudinal studies [157,158].

Overall, we rate the quality of the evidence *supporting* an association between traffic noise and markers of obesity, respectively, as “low”. This indicates that further research is very likely to have an important impact on our confidence in the estimate of effect and is likely to change the estimate.

### 3.6. Blood Pressure in Children

We evaluated eight studies investigating the impact of noise on children’s blood pressure [31,39,41,48,58,59,71,93,114,119,159]. Appendix B presents the separate risk of bias tables, and Appendix H presents the different GRADE tables (summarized in Table 7). Seven studies were cross-sectional; one study reported both the results of cross-sectional and longitudinal analyses. With the exception of the association between road traffic noise at school and systolic blood pressure, we observed positive but non-significant associations between exposure to road traffic noise and blood pressure (see Table 7). No combined exposure-response estimate could be computed from the studies on the impact of aircraft noise, since no quantitative results were provided in one of the studies. 

Overall, we rate the quality of the evidence *supporting* an association between traffic noise and blood pressure in children, as “very low”, indicating that any estimate of effect is very uncertain.

### 3.7. Wind Turbine Noise

Overall, we evaluated only three cross-sectional studies that investigated the impact of noise from wind turbines on the cardiovascular and metabolic systems [60,65,76,81,84,86,101]. Important limitations of these studies were the low response rates (two studies had response rates of less than 60%) and, the fact that in all studies the cardiovascular or metabolic endpoint was ascertained by questionnaire or interview. In these studies, we observed that an increase in wind turbine noise was associated with non-significant increases in self-reported hypertension and non-significant decreases in self-reported cardiovascular disease. For self-reported diabetes, the results appeared inconsistent.

Overall, we rate the quality of the studies *supporting* an association between exposure from wind turbine noise and adverse effects in the cardiovascular or metabolic system as “very low”, indicating that any estimate of effect is very uncertain.

## 4. Discussion

The current review shows that a large number of studies have investigated the impact of noise on the cardiovascular system, but applying the GRADE, the quality of the evidence is often rated as relatively low. This does *not* mean that exposure to noise has no effect on the cardiovascular system, but encourages further research to improve the quality of the evidence. After all, there is a strong biological plausibility that noise affects human health. Furthermore, in many of the evaluated studies, we observed statistically significant associations between noise and cardiovascular endpoints. The most robust were the effects of road traffic noise in relation to IHD. Combining the results of 7 longitudinal studies, revealed an RR of 1.08 (95% CI: 1.01–1.15) per 10 dB (L_DEN_) for the association between road traffic noise and the *incidence* of IHD. We rated the quality of the evidence from these longitudinal studies as high. Supporting evidence came from studies on the association between road traffic noise and the *prevalence* of IHD.

Several recent reviews have been published on cardiovascular effects of environmental noise exposure, which are described in detail in the full systematic review [1]. The quantitative results regarding exposure-response relationships following meta-analyses agree well with our review. However, most earlier reviews did not include a detailed quality assessment of individual studies.

This review also addressed the possible impact of noise on the metabolic system. In comparison with the studies on the impact of noise on the cardiovascular system, the number of available studies was rather limited. The results of these studies were not always consistent. In addition, the quality of the evidence was rather low. It is therefore, at this moment too early to draw definite conclusions with regard to the impact of noise on the metabolic system. 

## 5. Conclusions

The results of the current review shows that at this moment, not enough studies of good quality are available that investigated the impact of noise on the cardiovascular and metabolic system. The plausibility of an association calls for further efforts with improved research. In order to improve the quality of the existing evidence, more studies with a cohort or case-control design are needed.

In order to improve the quality of the existing evidence, we also recommend that more well designed studies on health effects in relation to exposure to wind turbines and rail traffic noise are set up and carried out.

## Figures and Tables

**Table 1:** The levels of quality of evidence of the GRADE system (source: [146,147]).

Quality of Evidence	Definition	Examples of When This is the Case
High	Further research is very unlikely to change our confidence in the estimate of effect	Several high-quality studies with consistent results
Moderate	Further research is likely to have an important impact on our confidence in the estimate of effect and may change the estimate	One high-quality study or several studies with some limitations
Low	Further research is very likely to have an important impact on our confidence in the estimate of effect and is likely to change the estimate	One or more studies with severe limitations
Very Low	Any estimate of effect is very uncertain	No direct research evidence
		One or more studies with very severe limitations

**Table 2:** Noise exposure and the risk of hypertension: summary of findings.

Noise Source	Outcome ^$^	Number of Study Design (s) *	RR per 10 dB (95% CI) ^†^	Number of Participants (Cases)	Quality of Evidence ^‡^
Air traffic	Prev	9 CS	1.05 (0.95–1.17)	60,121 (9487)	⊕⊕
	Inc	1 CO	1.00 (0.77–1.30)	4721 (1346)	⊕⊕
Road traffic	Prev	26 CS	1.05 (1.02–1.08) **	154,398 (18,957)	⊕
	Inc	1 CO	0.97 (0.90–1.05)	32,635 (3145)	⊕⊕
Rail traffic	Prev	5 CS	1.05 (0.88–1.26)	15,850 (2059)	⊕
	Inc	1 CO	0.96 (0.88–1.04)	7249 (3145)	⊕⊕
Wind turbine	Prev	3 CS	††	1830 (NR)	⊕

^$^ Outcome: Prev = prevalence of hypertension, Inc = incidence of hypertension; * CS = cross-sectional study, CO = cohort study; ^†^: RR = Relative risk per 10 decibel (dB change in noise level and its 95% confidence interval (CI) after aggregating the results of the evaluated studies. For air, road, –and, rail traffic, noise levels were expressed in L_DEN_. For wind turbines, noise levels are expressed in Sound Pressure Levels (SPL); ^‡^ GRADE Working Group Grades of Evidence: High quality (⊕⊕⊕⊕): Further research is very unlikely to change our confidence in the estimate of effect, Moderate Quality (⊕⊕⊕): Further research is likely to have an important impact on our confidence in the estimate of effect and may change the estimate, Low Quality (⊕⊕): Further research is very likely to have an important impact on our confidence in the estimate of effect and is likely to change the estimate, Very low quality (⊕): We are very uncertain about the estimate. ** The estimate for the association between road traffic noise and the prevalence of hypertension is based on 47 estimates derived from 26 studies. †† We decided not to aggregate the results of the three studies on the impact of wind turbine noise, since too many parameters were unknown and/or unclear. NR = Not Reported.

**Table 3:** Noise exposure and the risk of IHD: summary of findings.

Noise Source	Outcome ^$^	Number of Study Design (s) *	RR ^†^ per 10 dB (95% CI)	Participants (Cases)	Quality of Evidence ^‡^
Air traffic	Prev	2 CS	1.07 (0.94–1.23)	14,098 (340)	⊕
	Inc	2 ECO	1.09 (1.04–1.15)	9,619,082 (158,977)	⊕
	Mort	2 ECO	1.04 (0.97–1.12)	3,897,645 (26,066)	⊕
		1 CO	1.04 (0.98–1.11)	4,580,311(15,532)	⊕⊕
Road traffic	Prev	8 CS	*1.24 (1.08–1.42)*	25,682 (1614)	⊕⊕
	Inc	1 ECO	1.12 (0.85–1.48)	262,830 (418)	⊕
		3 CO, 4CC	*1.08 (1.01–1.15)*	67,224 (7033)	⊕⊕⊕⊕
	Mort	1 CC, 2 CO	1.05 (0.97–1.13)	532,268 (6884)	⊕⊕⊕
Rail traffic	Prev	4 CS	1.18 (0.82–1.68)	13,241 (283)	⊕

^$^ Outcome: Prev = prevalence of IHD, Inc = incidence of IHD, Mort = mortality due to IHD; * ECO = ecological study, CS = cross-sectional study, CC = case-control study, CO = cohort study; ^†^: RR = Relative Risk per 10 decibel (dB change in noise level, 95% CI = 95% Confidence Interval. For air, road –and, rail traffic, noise levels are expressed in L_DEN_.; ^‡^ GRADE Working Group Grades of Evidence: High quality (⊕⊕⊕⊕): Further research is very unlikely to change our confidence in the estimate of effect, Moderate Quality (⊕⊕⊕): Further research is likely to have an important impact on our confidence in the estimate of effect and may change the estimate, Low Quality (⊕⊕): Further research is very likely to have an important impact on our confidence in the estimate of effect and is likely to change the estimate, Very low quality (⊕): We are very uncertain about the estimate.

**Table 4:** Noise exposure and the risk of stroke: summary of findings.

Noise Source	Outcome ^$^	Number of Study Design (s) *	RR ^†^ per 10 dB (95% CI)	Participants (Cases)	Quality of Evidence ^‡^
Air traffic	Prev	2 CS	1.02 (0.80–1.28)	14,098 (151)	⊕
	Inc	2 ECO	1.05 (0.96–1.15)	9,619,082 (97,949)	⊕
	Mort	2 ECO	1.07 (0.98–1.17)	3,897,645 (12,086)	⊕
		1 CO	0.99 (0.94–1.04)	4,580,311 (25,231)	⊕⊕⊕
Road traffic	Prev	2 CS	1.00 (0.91–1.10)	14,098 (151)	⊕
	Inc	1 CO	*1.14 (1.03–1.25)*	51,485 (1881)	⊕⊕⊕
	Mort	3 CO	0.87 (0.71–1.06)	581,517 (2634)	⊕⊕⊕
Rail traffic	Prev	1 CS	1.07 (0.92–1.25)	9365 (89)	⊕

^$^ Outcome: Prev = prevalence of stroke, Inc = incidence of stroke, Mort = mortality due to stroke; * ECO = ecological study, CS = cross-sectional study, CO = cohort study; ^†^: RR = Relative risk per 10 decibel (dB change in noise level, 95% CI = 95% Confidence Interval. The noise levels are expressed in L_DEN_; ^‡^ GRADE Working Group Grades of Evidence: High quality (⊕⊕⊕⊕): Further research is very unlikely to change our confidence in the estimate of effect, Moderate Quality (⊕⊕⊕): Further research is likely to have an important impact on our confidence in the estimate of effect and may change the estimate, Low Quality (⊕⊕): Further research is very likely to have an important impact on our confidence in the estimate of effect and is likely to change the estimate, Very low quality (⊕): We are very uncertain about the estimate.

**Table 5:** Noise exposure and the risk of diabetes: summary of findings.

Noise Source	Outcome ^$^	Number of Study Design (s) *	RR ^†^ per 10 dB (95% CI)	Participants (Cases)	Quality of Evidence ^‡^
Air traffic	Prev	1 CS	1.01 (0.78–1.31)	9365 (89)	⊕
	Inc	1 CO	0.99 (0.47–2.09)	5156 (159)	⊕⊕
Road traffic	Prev	2 CS	- ^#^	11,460 (242)	⊕
	Inc	1 CO	*1.08 (1.02–1.14)*	57,053 (2752)	⊕⊕⊕
Rail traffic	Prev	1 CS	*0.21 (0.05–0.82)*	9365 (89)	⊕
	Inc	1 CO	0.97 (0.89–1.05)	57,053 (2752)	⊕⊕⊕
Wind turbine	Prev	3 CS	**	1830 (NR)	⊕

^$^ Outcome: Prev = prevalence of diabetes, Inc = incidence of diabetes; * CS = cross-sectional study, CO = cohort study; ^†^ RR = Relative risk per 10 decibel (dB change in noise level, 95% CI = 95% Confidence Interval. For air, road –and, rail traffic, noise levels are expressed in L_DEN_. For wind turbines, noise levels were expressed in Sound Pressure Levels (SPL); ^‡^ GRADE Working Group Grades of Evidence: High quality (⊕⊕⊕⊕): Further research is very unlikely to change our confidence in the estimate of effect, Moderate Quality (⊕⊕⊕): Further research is likely to have an important impact on our confidence in the estimate of effect and may change the estimate, Low Quality (⊕⊕): Further research is very likely to have an important impact on our confidence in the estimate of effect and is likely to change the estimate, Very low quality (⊕): We are very uncertain about the estimate; ^#^ the data from one cross-sectional study were not included in the table since they were based on a secondary analysis with important information lacking. ** We decided not to aggregate the results of the three studies on the impact of wind turbine noise, since too many parameters were unknown and/or unclear; NR = Not Reported.

**Table 6:** Noise exposure and the risk of obesity: summary of findings.

Noise Source	Outcome	Number of Study Design (s) *	Change per 10 dB (95% CI) ^†^	Participants	Quality of Evidence ^‡^
Air traffic	Change in BMI (kg/m^2^)	1 CO	0.14 (−0.18–0.45)	5156	⊕⊕
	Change in waist circumference (cm)	1 CO	*3.46 (2.13–4.77)*	5156	⊕⊕⊕
Road traffic	Change in BMI (kg/m^2^)	3 CS	0.03 (−0.10–0.15)	71,431	⊕
	Change in waist circumference (cm)	3 CS	0.17 (−0.06–0.40)	71,431	⊕
Rail traffic	Change in BMI (kg/m^2^)	2 CS	- **	57,531	⊕
	Change in waist circumference (cm)	2 CS	- **	57,531	⊕⊕

* CS = cross-sectional study, CO = cohort study; ^†^ 95% CI = 95% Confidence Interval. Noise levels are expressed in L_DEN_; ^‡^ GRADE Working Group Grades of Evidence: High quality (⊕⊕⊕⊕): Further research is very unlikely to change our confidence in the estimate of effect, Moderate Quality (⊕⊕⊕): Further research is likely to have an important impact on our confidence in the estimate of effect and may change the estimate, Low Quality (⊕⊕): Further research is very likely to have an important impact on our confidence in the estimate of effect and is likely to change the estimate, Very low quality (⊕): We are very uncertain about the estimate. ** We decided not to aggregate the results of the studies on the impact of rail traffic noise, since not all parameters were available to assess a change in BMI or waist circumference per 10 dB; dB = Decibel, BMI = Body Mass Index.

**Table 7:** Noise exposure and the impact on children’s blood pressure: summary of findings.

Noise Source	Setting	Outcome	Number of Study Design (s) *	Change in Blood Pressure (mmHg) per 10 dB (95% CI) ^†^	Participants	Quality of Evidence ^‡^
Air traffic	School	Systolic blood pressure (mmHg)	2 CS	-	2013	⊕
		Diastolic blood pressure (mmHg)	2 CS	-	2013	⊕
	Home	Systolic blood pressure (mmHg)	2 CS	-	2013	⊕
		Diastolic blood pressure (mmHg)	2 CS	-	2013	⊕
Road traffic	School	Systolic blood pressure (mmHg)	5 CS	−0.60 (−1.51–0.30)	4520	⊕
		Diastolic blood pressure (mmHg)	5 CS	0.46 (−0.60–1.53)	4520	⊕
	Home	Systolic blood pressure (mmHg)	6 CS	0.08 (−0.48–0.64)	4197	⊕
		Diastolic blood pressure (mmHg)	6 CS	0.47 (−0.30–1.24)	4197	⊕

* CS = Cross-sectional study; ^†^ 95% CI: 95% confidence interval. Blood pressure is expressed in millimeters of mercury (mmHg). Noise levels are expressed in L_DEN_; ^‡^ GRADE Working Group Grades of Evidence: High quality (⊕⊕⊕⊕): Further research is very unlikely to change our confidence in the estimate of effect, Moderate Quality (⊕⊕⊕): Further research is likely to have an important impact on our confidence in the estimate of effect and may change the estimate, Low Quality (⊕⊕): Further research is very likely to have an important impact on our confidence in the estimate of effect and is likely to change the estimate, Very low quality (⊕): We are very uncertain about the estimate; mmHg: millimeters of mercury.

## Appendix A. Applied Search Profiles

In order to identify “Observational studies such as ecological studies, cross-sectional studies, case control studies or cohort studies involving the association between aircraft and/or rail traffic noise exposure and hypertension and/or high blood pressure, and/or ischemic heart disease (including angina pectoris and/or myocardial infarction) in adults published from 2000 until October 2014 with no language restriction”, the following search profiles were applied in:

MEDLINE 1950 to present, MEDLINE In-Process & Other Non-Indexed Citations 20141021

1 ((rail* or aircraft or airport* or air traffic*) adj5 noise.tw. (504)
2 Aircraft/or Airports/or Railroads/(9486)
3 *Transportation/(3419)
4 (rail* or aircraft or airport* or air traffic.tw. (11,558)
5 *Noise/(10,029)
6 Noise, transportation/(1017)
7 exp Blood pressure/(254,113)
8 exp Hypertension/(217,361)
9 Myocardial ischemia/(33,403)
10 exp Cardiovascular diseases/or exp Vascular diseases/or exp Heart diseases/(1,944,605)
11 (hypertension or blood pressure.tw. (445,550)
12 (isch?emic heart disease* or coronary heart disease* or angina pectoris or myocard* infarct*or cardiovascular disease* or heart disease*).tw. (368,878)
13 (1 or 2 or (3 and 4)) and (1 or 5 or 6) (860)
14 13 and (7 or 8 or 9 or 10 or 11 or 12) (119)
15 14 not child*.ti. (112)
16 limit 15 to yr = 2000 − current (83)

Scopus, 20141022

((TITLE-ABS-KEY((rail* OR aircraft OR airport* OR air-traffic*) W/5 noise) AND (TITLE-ABS-KEY(hypertension OR blood-pressure OR ischemic-heart-disease* OR coronary-heart-disease* OR angina-pectoris OR myocard*-infarct* OR cardiovascular-disease* OR heart-disease*)) AND PUBYEAR > 1999) AND NOT (TITLE(child*))

In order to identify “Observational studies such as ecological studies, cross-sectional studies, case-control studies or cohort studies involving the association between aircraft and/or rail traffic and/or road traffic noise exposure and stroke and/or diabetes type II, and/or obesity in adults, published until October 2014 with no language restriction”, the following search profiles were applied in:

Medline 20141023 MEDLINE 1950 to present, MEDLINE In-Process & Other Non-Indexed Citations

1 ((rail* or aircraft or airport* or road* or traffic* or automobile* or vehicle*) adj5 noise.tw.(1188)
2 exp *Transportation/(35,715)
3 Aircraft/or Airports/or Railroads/or Motor Vehicles/(12,387)
4 *Noise/(10,039)
5 Noise, transportation/(1023)
6 (1 or 2 or 3) and (1 or 4 or 5) (1774)
7 exp Cerebrovascular disorders/(290,152)
8 exp Diabetes Mellitus/(328,383)
9 exp Obesity/or exp Overweight/or exp Body Mass Index/(208,810)
10 (stroke or cerebrovascular* or cva or brain vascular accident* or brain vascular disorder*).tw. (187,910)
11 (diabetes or obesit* or overweight or bmi or body mass index).tw. (556,663)
12 7 or 8 or 9 or 10 or 11 (1,065,975)
13 6 and 12 (54)
14 13 not child*.ti. (51)
15 limit 14 to yr = 2000 − current (47)

Scopus 20141023

((TITLE-ABS-KEY((rail* OR aircraft OR airport* OR road* OR traffic* OR automobile* OR vehicle*) W/1 noise) AND (TITLE-ABS-KEY(stroke OR cerebrovascular OR cva OR brain-vascular OR diabetes OR obesit* OR overweight OR bmi OR body-mass-index)) AND PUBYEAR > 1999) AND NOT (TITLE(child*))

In order to identify “Observational studies such as ecological studies, cross-sectional studies, case control studies or cohort studies involving the association between road traffic noise exposure and hypertension and/or high blood pressure published from 2010 until October 2014 with no language restriction”, the following search profiles were applied in:

Medline 20141017 MEDLINE 1950 to present, MEDLINE In-Process & Other Non-Indexed Citations

1 ((road* or traffic* or automobile* or vehicle* or motor cycle* or motorcycle* or transport*) adj5 noise.tw.(993)
2 exp *Transportation/(35,698)
3 Motor Vehicles/(2962)
4 *Noise/(10,029)
5 Noise, transportation/(1017)
6 (1 or 2 or 3) and (1 or 4 or 5) (1714)
7 exp Blood pressure/(254,113)
8 exp Hypertension/(217,361)
9 (blood pressure or hypertension).tw. (445,404)
10 6 and (7 or 8 or 9) (134)
11 10 not child*.ti. (120)
12 limit 11 to yr = 2010 − current (46)

PubMed 20141024

((traffic*[ti] OR road*[ti] OR automobile*[ti] OR vehicle*[ti] OR motorcycle*[ti] OR transport*[ti]) AND noise[ti]

Scopus 20141024

(TITLE-ABS-KEY((rail* OR aircraft OR airport* OR road* OR traffic* OR automobile* OR vehicle*) W/1 noise) AND (TITLE-ABS-KEY(hypertension OR blood-pressure) AND PUBYEAR > 2009 AND NOT TITLE(child*)

In order to identify “Observational studies such as ecological studies, cross-sectional studies, case-control studies or cohort studies involving the association between road, rail and air traffic noise exposure and blood pressure in children published until October 2014 without any language restriction”, the following search profiles were applied in:

Medline 20141017 MEDLINE 1950 to present, MEDLINE In-Process & Other Non-Indexed Citations

1 ((rail* or aircraft or airport* or road* or traffic or automobile* or vehicle*) adj5 noise.tw. (1185)
2 exp *Transportation/(35,698)
3 Aircraft/or Airports/or Railroads/or Motor Vehicles/(12,379)
4 *Noise/(10,029)
5 Noise, transportation/(1017)
6 (1 or 2 or 3) and (1 or 4 or 5) (1770)
7 exp Blood pressure/(254,113)
8 exp Hypertension/(217,361)
9 (blood pressure or hypertension).tw. (445,404)
10 6 and (7 or 8 or 9) (144)
11 10 and (child* or infant* or adolescent*).mp. (43)

Scopus 20141024

TITLE-ABS-KEY((rail* OR aircraft OR airport* OR road* OR traffic* OR automobile* OR vehicle*) W/1 noise AND TITLE-ABS-KEY(blood-pressure OR hypertension) AND TITLE-ABS-KEY(child* OR infant* OR adolescent*)

In order to identify “Observational studies such as ecological studies, cross-sectional studies, case-control studies or cohort studies involving the association between audible noise (greater than 20 Hz) and infrasound and low-frequency noise (less than 20 Hz) from wind turbines or wind farms and blood pressure and/or cardiovascular disease published from October 2012 until October 2014 without any language restriction”, the following search profiles were applied in:

PubMed 20141024

(((((wind turbine* OR wind farm*[Title/Abstract]))) AND ((noise[MeSH Terms]) OR noise[Title/Abstract]))) AND (((health*[Title/Abstract]) OR blood pressure OR cardiovascular)) 2012–current

Medline 20141027 MEDLINE 1950 to present, MEDLINE In-Process & Other Non-Indexed Citations

1 ((wind adj3 turbine*) or (wind adj3 farm*) or windturbine* or windfarm*).tw. (271)
2 Wind/(2794)
3 Renewable energy/(273)
4 Power Plants/(5234)
5 Electric Power Supplies/(4979)
6 Energy-Generating Resources/(1684)
7 2 and (3 or 4 or 5 or 6) (183)
8 1 or 7 (362)
9 Noise/or Sound/(26,842)
10 (infrasound* or noise or low frequenc*).tw. (131,959)
11 (blood pressure or cardiovascular).tw. (474,959)
12 Blood Pressure/(243,394)
13 Cardiovascular Physiological Phenomena/or Cardiovascular Diseases/or Cardiovascular System/(129,880)
14 health*.ti. (532,337)
15 8 and (9 or 10) and (11 or 12 or 13 or 14) (19)
16 limit 15 to yr = 2012–current (14)

Scopus 20141027

TITLE-ABS-KEY((wind W/3 turbine*) OR windturbine* OR (wind W/3 farm*) OR windfarm*) AND TITLE-ABS-KEY(noise OR infrasound* OR low-frequenc*) AND (TITLE-ABS-KEY(blood-pressure OR cardiovascular*) OR TITLE(health*) OR KEY(health*)) AND PUBYEAR > 2011

Embase and SciSearch:

same search profile used as in Medline.

## Appendix B. Risk of Bias

This appendix presents the risk of bias tables. They are presented per exposure outcome combination. An extensive description and the reasoning behind these tables can be found in Chapters 4–9 of the complete review.

**Table A1:** Reviewer’s judgement about risk of bias for each of the studies on aircraft noise and hypertension that were selected for data extraction.

Study [Ref.]	Bias Due to Exposure Assessment	Bias Due to Confounding *	Bias Due to Selection of Participants ^†^	Bias Due to Health Outcome Assessment	Bias Due to Not Blinded Outcome Assessment	Total Risk of Bias
SDPP [73,78,91,106]	Low	Low	High	Low	Low	Low
HYENA [50,61,62,83,85,98,99]	Low	Low	High	Low	High	High
SEHS [112]	Low	Low	Low	High	Low	Low
DEBATS-pilot [46]	Low	Low	High	Low	Unclear	High
DEBATS-main [26]	Low	Low	Unclear	Low	Unclear	Unclear
AWACS [28]	Low	Low	High	High	Low	High
Okinawa [40,102,113]	High	Low	Unclear	Low	Low	High
Knipschild-1 [133,134]	High	High	High	Low	Low	High
SERA [74]	Low	Low	High	Low	Unclear	High
GES-2 [94,95,105]	Low	Low	High	High	Low	High
GES-3 [94,95,105]	Low	Low	High	High	Low	High
SPANDAU [97,109,110]	Low	Low	Low	High	Low	Low

* In order to score “low”, the study should contain information that can be used to derive effect estimates that are at least adjusted for age and sex; ^†^ In order to score “low” participants had to be randomly sampled from a known population and the response rate of the study had to be higher than 60% (cross-sectional studies) and attrition rate is less than 20% (follow-up studies).

**Table A2:** Reviewer’s judgement about risk of bias for each of the studies on road traffic noise and hypertension that were selected for data extraction.

Study [Ref.]	Bias Due to Exposure Assessment	Bias Due to Confounding *	Bias Due to Selection of Participants ^†^	Bias Due to Health Outcome Assessment	Bias Due to Not Blinded Outcome Assessment	Total Risk of Bias
Amsterdam [132]	High	Low	Low	Low	Low	Low
Caerphilly [130,131]	High	High	Low	Low	Low	High
Luebeck [126,127]	High	Low	Low	Low	Unclear	High
BCC3 [118,120,123]	Low	Low	High	High	Low	High
SHEEP [75]	Low	Low	Low	Low	Low	Low
Tokyo [117]	Unclear	Low	Low	High	Unclear	Unclear
StockholmRoad [92]	Low	High	Low	High	Low	High
Groningen [88,89]	Low	Low	High	High	Low	High
PREVEND [88,89]	Low	Low	High	Low	Low	Low
UIT1 [135]	Low	High	Low	High	Unclear	High
SPANDAU [97,109,110]	Low	Low	Low	High	Low	Low
Skane-1 [96]	Low	Low	High	High	Unclear	High
Lerum [80]	Low	Low	Low	High	High	High
Skane-2 [77]	Low	Low	Low	High	Low	Low
BBT-1 (phone [82,135]	Low	Low	Low	High	Unclear	High
BBT-2 (face-to-face [82,135]	Low	Low	Low	High	Unclear	High
HYENA [50,61,62,83,85,98,99]	Low	Low	High	Low	High	High
KORA [37,49]	Low	Low	Low	Low	Low	Low
Berlin-IV [36,149]	Low	Low	High	Low	Low	Low
Taiwan [35,70]	High	Low	Unclear	High	Unclear	High
REGICOR [32,33,43,68]	Low	Low	Low	Low	Low	Low
Heinz-Nixdorf Recall Study [67]	Low	Low	Low	Low	Low	Low
Oslo Health Study [30,66]	Low	Low	Low	Low	Low	Low
DCH [51,63]	Low	Low	High	High	Low	High
SAPALDIA-2 [55,57]	Low	Low	Low	High	Low	Low
Roadside [56]	Low	High	High	High	Low	High
ALPNAP [82,90,135]	Low	Low	High	High	Unclear	High
AWACS [28]	Low	Low	High	High	Low	High

* In order to score “low”, the study should contain information that can be used to derive effect estimates that are at least adjusted for age and sex; ^†^ In order to score “low” participants had to be randomly sampled from a known population and the response rate of the study had to be higher than 60%.

**Table A3:** Reviewer’s judgement about risk of bias for each of the studies on rail traffic noise and hypertension that were selected for data extraction.

Study [Ref.]	Bias Due to Exposure Assessment	Bias Due to Confounding *	Bias Due to Selection of Participants ^†^	Bias Due to Health Outcome Assessment	Bias Due to Not Blinded Outcome Assessment	Total Risk of Bias
Lerum [80]	Low	Low	Low	High	High	High
AWACS [28]	Low	Low	High	High	Low	High
Roadside [56]	Low	High	High	High	Low	High
DCH [51,63]	Low	Low	High	High	Low	High
SAPALDIA-2 [55,57]	Unclear	Low	Low	High	Low	High
ALPNAP [82,90,135]	Low	Low	High	High	Unclear	High
BBT-1 [82,135]	Low	Low	Low	High	Unclear	High
BBT-2 [82,135]	Low	Low	Low	High	Unclear	High

* In order to score “low”, the study should contain information that can be used to derive effect estimates that are at least adjusted for age and sex; ^†^ In order to score “low” participants had to be randomly sampled from a known population and the response rate of the study had to be higher than 60%.

**Table A4:** Reviewer’s judgement about risk of bias for each of the studies on noise from wind turbines and hypertension that were selected for data extraction.

Study [Ref.]	Bias Due to Exposure Assessment	Bias Due to Confounding *	Bias Due to Selection of Participants ^†^	Bias Due to Health Outcome Assessment	Bias Due to Not Blinded Outcome Assessment	Total Risk of Bias
NL-07 [60,65,76,84]	High	Low	High	High	Low	High
SWE-00 [65,81,101]	High	Low	Low	High	Low	High
SWE-05 [65,81,86]	High	Low	High	High	Low	High

* In order to score “low”, the study should contain information that can be used to derive effect estimates that are at least adjusted for age and sex; ^†^ In order to score “low” participants had to be randomly sampled from a known population and the response rate of the study had to be higher than 60%.

**Table A5:** Reviewer’s judgement about risk of bias for each of the studies on aircraft noise and IHD that were selected for data extraction.

Study [Ref.]	Bias Due to Exposure Assessment	Bias Due to Confounding *	Bias Due to Selection of Participants ^†^	Bias Due to Health Outcome Assessment	Bias Due to Not Blinded Outcome Assessment	Total Risk of Bias
HYENA [44,45,50,61,62,69,83,85,98,99]	Low	Low	High	High	High	High
USAairports [47]	High	High	Low	Low	Low	High
SPANDAU [97,109,110]	Low	High	Low	High	Low	High
LSAS [42]	High	Unclear	Low	Low	Low	High
SNC [72]	Unclear	High	Low	Low	Low	High
AWACS-1 [28]	Low	Low	High	High	Low	High
AWACS-2 [28]	Unclear	High	Low	Low	Low	High
IVEM [124,128,129]	High	High	Low	Low	Low	High

* In order to score “low”, the study should contain information that can be used to derive effect estimates that are at least adjusted for age, sex, and smoking; ^†^ In order to score “low” participants had to be randomly sampled from a known population and the response rate of the study had to be higher than 60%. Studies with a purposeful sample also scored “low”.

**Table A6:** Reviewer’s judgement about risk of bias for each of the studies on road traffic noise and IHD that were selected for data extraction.

Study [Ref.]	Bias Due to Exposure Assessment	Bias Due to Confounding *	Bias Due to Selection of Participants ^†^	Bias Due to Health Outcome Assessment	Bias Due to Not Blinded Outcome Assessment	Total Risk of Bias
Caerphilly-a [122,125,130,131]	High	High	Low	Low	Low	High
Caerphilly-b [111,115,122,125,130,131]	High	Low	Low	Low	Low	Low
Speedwell-a [121,122,125,131]	High	High	Low	Low	Low	High
Speedwell-b [111,115,121,122,125,131]	High	Low	Low	Low	Low	Low
SPANDAU [97,109,110]	Low	High	Low	High	Low	High
ALPNAP [82,90,135]	Low	Low	High	High	Unclear	High
NAROMI [100,107]	Low	Low	Low	Low	Low	Low
BCC1 [118,120,123]	Low	Low	Low	Low	Low	Low
BCC2 [118,120,123]	Low	Low	Low	Low	Low	Low
BCC3 [118,120,123]	Low	Low	Low	High	High	High
Kaunus-1 [87,103]	High	High	Low	Low	Low	High
BBT-Phone [82,135]	Low	High	Low	High	Unclear	High
BBT-Face [82,135]	Low	High	Low	High	Unclear	High
IVEM [124,128,129]	High	High	Low	Low	Low	High
SHEEP [75]	Low	Low	Low	Low	Low	Low
NCSDC [79]	Low	Low	Low	Low	Low	Low
AWACS1 [28]	Low	Low	High	High	Low	High
HYENA [44,45,50,61,62,69,83,85,98,99]	Low	Low	High	High	High	High
DCH [52,53]	Low	Low	Low	Low	Low	Low
Canada1 [54]	Low	High	Low	Low	Low	Low

* In order to score “low”, the study should contain information that can be used to derive effect estimates that are at least adjusted for age, sex, and smoking; ^†^ In order to score “low”, participants had to be randomly sampled from a known population and the response rate of the study had to be higher than 60%. Studies with a purposeful sample also scored “low”.

**Table A7:** Reviewer’s judgement about risk of bias for each of the studies on rail traffic noise and IHD that were selected for data extraction.

Study [Ref.]	Bias Due to Exposure Assessment	Bias Due to Confounding *	Bias Due to Selection of Participants ^†^	Bias Due to Health Outcome Assessment	Bias Due to not Blinded Outcome Assessment	Total Risk of Bias
BBT-1 [82,135]	Low	High	Low	High	Unclear	High
BBT-2 [82,135]	Low	High	Low	High	Unclear	High
ALPNAP [82,90,135]	Low	Low	High	High	Unclear	High
AWACS [28]	Low	Low	High	High	Low	High

* In order to score “low”, the study should contain information that can be used to derive effect estimates that are at least adjusted for age, sex, and smoking; ^†^ In order to score “low”, participants had to be randomly sampled from a known population and the response rate of the study had to be higher than 60%. Studies with a purposeful sample also scored “low”.

**Table A8:** Risk of bias: reviewer’s judgements about each risk of bias item for each of the six studies on the association between aircraft noise and stroke that were selected for data extraction.

Study [Ref.]	Bias Due to Exposure Assessment	Bias Due to Confounding *	Bias Due to Selection of Participants ^†^	Bias Due to Health Outcome Assessment	Bias Due to Not Blinded Outcome Assessment	Total Risk of Bias
HYENA [44,45,50,61,62,69,83,85,98,99]	Low	Low	High	High	High	High
LSAS [42]	High	High	Low	Low	Low	High
SNC [72]	Unclear	High	Low	Low	Low	High
AWACS-1 [28]	Low	Low	High	High	Low	High
AWACS-2 [28]	Unclear	High	Low	Low	Low	High
USAairports [47]	High	High	Low	Low	Low	High

* In order to score “low”, the study should contain information that can be used to derive effect estimates that are at least adjusted for age, sex, and smoking; ^†^ In order to score “low”, participants had to be randomly sampled from a known population and the response rate of the study had to be higher than 60%. Studies with a purposeful sample also scored “low”.

**Table A9:** Reviewer’s judgement about risk of bias for each of the studies on road traffic noise and stroke that were selected for data-extraction.

Study [Ref.]	Bias Due to Exposure Assessment	Bias Due to Confounding *	Bias Due to Selection of Participants ^†^	Bias Due to Health Outcome Assessment	Bias Due to Not Blinded Outcome Assessment	Total Risk of Bias
HYENA [44,45,50,61,62,69,83,85,98,99]	Low	Low	High	High	High	High
NCSDC [79]	Low	Low	Low	Low	Low	Low
DCH [27,52,64]	Low	Low	Low	Low	Low	Low
AWACS1 [28]	Low	Low	High	High	Low	High
Canada1 [54]	Low	High	Low	Low	Low	Low

* In order to score “low”, the study should contain information that can be used to derive effect estimates that are at least adjusted for age, sex, and smoking; ^†^ In order to score “low” participants had to be randomly sampled from a known population and the response rate of the study had to be higher than 60%. Studies with a purposeful sample also scored “low”.

Only the AWACS1 study [28] investigated the impact of rail traffic noise on stroke. See Table A9 for the quality assessment.

**Table A10:** Reviewer’s judgement on risk of bias in studies on aircraft noise and diabetes.

Study	Bias Due to Exposure Assessment	Bias Due to Confounding *	Bias Due to Selection of Participants ^†^	Bias Due to Health Outcome Assessment	Bias Due to Not Blinded Outcome Assessment	Total Risk of Bias
SDPP [34]	Low	Low	High	Low	Low	Low
AWACS-1 [28]	Low	Low	High	High	Low	High

* In order to score “low”, the study should contain information that can be used to derive effect estimates that are at least adjusted for age, sex, and smoking; ^†^ In order to score “low” participants had to be randomly sampled from a known population and the response rate of the study had to be higher than 60%. Studies with a purposeful sample also scored “low”.

**Table A11:** Reviewer’s judgement on risk of bias in studies on road traffic noise and diabetes.

Study	Bias Due to Exposure Assessment	Bias Due to Confounding *	Bias Due to Selection of Participants ^†^	Bias Due to Health Outcome Assessment	Bias Due to Not Blinded Outcome Assessment	Total Risk of Bias
SHEEP [75]	Low	Low	Low	High	Low	Low
DCH [38]	Low	Low	Low	Low	Low	Low
AWACS1 [28]	Low	Low	High	High	Low	High

* In order to score “low”, the study should contain information that can be used to derive effect estimates that are at least adjusted for age, sex, and smoking; ^†^ In order to score “low” participants had to be randomly sampled from a known population and the response rate of the study had to be higher than 60%. Studies with a purposeful sample also scored “low”.

Table A4 also presents the results of the evaluation of the quality of the studies that investigated the association between audible noise (greater than 20 Hz) from wind turbines and self-reported diabetes [60,65,76,81,84,86,101].

Table A10 also presents the results of the evaluation of the quality of the study that investigated the association between aircraft noise and obesity [34].

Table A11 also presents the results of the evaluation of the quality of the studies that assessed railway noise and diabetes: DCH [38], AWACS1 [28].

Table A12 also presents the results of the evaluation of the quality of the two studies that investigated the association between railway noise and obesity [136,155].

**Table A12:** Reviewer’s judgement on risk of bias in studies on road traffic noise and obesity.

Study	Bias Due to Exposure Assessment	Bias Due to Confounding *	Bias Due to Selection of Participants ^†^	Bias Due to Health Outcome Assessment	Bias Due to not Blinded Outcome Assessment	Total Risk of Bias
HUBRO [30,156]	Low	Low	High	Low	Low	Low
SDPP [155]	Low	Low	High	Low	Low	Low
DCH [136]	Low	Low	High	Low	Low	Low

* In order to score “low”, the study should contain information that can be used to derive effect estimates that are at least adjusted for age, sex, and smoking; ^†^ In order to score “low” participants had to be randomly sampled from a known population and the response rate of the study had to be higher than 60%. Studies with a purposeful sample also scored “low”.

**Table A13:** Risk of bias: reviewer’s judgements on risk of bias in studies on noise and children’s blood pressure.

Study	Bias Due to Exposure Assessment	Bias Due to Confounding *	Bias Due to Selection of Participants ^†^	Bias Due to Health Outcome Assessment	Bias Due to not Blinded Outcome Assessment	Total Risk of Bias
RANCH [58,93]	Unclear	Low	High	Unclear	Unclear	High
ICCBP-a [114,159]	Low	Low	High	Unclear	Unclear	High
ICCBP-b [114]	Low	Low	High	Unclear	Unclear	High
PIAMA [48]	Unclear	Low	High	Unclear	Low	High
GINIplus [31,41]	Unclear	Low	High	Unclear	Low	High
LISAplus [31,41]	Unclear	Low	High	Unclear	Low	High
BELGRADE1 [39]	High	Low	High	Unclear	Unclear	High
REGECOVA [119]	High	High	Low	Unclear	Unclear	High
USA1 [59,71]	High	High	Low	Unclear	Unclear	High

* In order to score “low” the study should contain information that can be used to derive effect estimates that are at least adjusted for age and sex. ^†^ In order to score “low”, participants had to be randomly sampled from a known population and the response rate of the study had to be higher than 60%. An additional condition for cohort studies was that the attrition rate had to be at least 20%.

## Appendix C. Summary of Findings Tables Dealing with Studies on the Impact of Noise on Hypertension

This appendix presents the summary of findings tables dealing with the studies on the impact of noise on hypertension. An extensive description and the reasoning behind these tables can be found in the complete review in Section 11.1.

**Table A14:** Summary of findings table for the association between aircraft noise exposure and the prevalence of hypertension.

Question	Does Exposure to Aircraft Noise Increase the Risk of Hypertension			
**People**	Adult population (men and women)			
**Setting**	Residential setting: people living in cities (general population) located around airports in Europe and Japan			
**Outcome**	The prevalence of hypertension			
**Summary of findings**	**RR per 10 dB increase in aircraft noise level (L_DEN_)**		1.05 (95% CI: 0.95–1.17) per 10 dB	
	**Number of participants (^#^ evaluated studies)**		60,121 (9)	
	**Number of cases**		9487	
			**Rating**	**Adjustment to rating**
**Quality assessment**	**Starting rating**		9 cross-sectional studies ^a^	2 (low)
	**Factors decreasing confidence**	**Risk of bias**	Serious ^b^	Downgrading
		**Inconsistency**	Serious ^c^	Downgrading
		**Indirectness**	None ^d^	No downgrading
		**Imprecision**	None ^e^	No downgrading
		**Publication bias**	None ^f^	No downgrading
	**Factors increasing confidence**	**Strength of association**	Small ^g^	No upgrading
		**Exposure-response gradient**	Non-significant exposure-response gradient ^g^	Upgrading
		**Possible confounding**	No serious bias ^h^	Upgrading
	**Overall judgement of quality of evidence**			0 (low)

^a^ Since only cross-sectional studies were available, we started with a grading of “low” (2); ^b^ Methods used to select the population: In six studies, the participants were randomly selected, taking into account aircraft noise exposure; three studies were originally not designed to investigate the impact of aircraft noise exposure, but still participants were randomly selected. In six studies, participants were probably not aware of the fact that they participated in a study investigating the impact of noise; for three studies, this was unclear. For one study, it was likely that participants were aware of the fact that they participated in a study investigating the impact of noise. In six studies, response rates were below 60%; for two studies, the response rate was unclear and only in one study response rate was higher than 60%; ^c^ Results across studies differed in magnitude and direction of effect estimates (see Figure 4.1 of the complete review). This was confirmed by the results of the heterogeneity analyses, demonstrating moderate heterogeneity (I^2^_residual_ = 72.1%); ^d^ The studies assessed population, exposure, and outcome of interest; ^e^ We considered the results to be precise, since the number of participants and the number of cases was large enough. The 95% confidence interval was sufficiently narrow; ^f^ There was little reason to believe that there is major publication bias or small study bias (see also Figure 4.2). The Egger test did not provide evidence for small-study effects; ^g^ Most studies found that the risk of hypertension increased when aircraft noise level increased (RR per 10 dB > 1). There was evidence of a non-significant exposure-response gradient: After aggregating the results of the evaluated studies, we found a non-significant effect size of 1.05 per 10 dB. The noise range of the studies under evaluation was 35–75 dB. This means that if air traffic noise level increases from 35 to 75 dB, the RR = 1.22. We found indications for an effect of exposure duration: The effect estimates turned out to be larger for the sample that lived for a longer period in the same house; ^h^ We did not find evidence that suggests that possible residual confounders or biases would reduce our effect estimate.

**Table A15:** Summary of findings table for the association between road traffic noise exposure and the prevalence of hypertension.

Question	Does Exposure to Road Traffic Noise Increase the Risk of Hypertension			
**People**	Adult population (men and women)			
**Setting**	Residential setting: people living several cities in Europe			
**Outcome**	The prevalence of hypertension			
**Summary of findings**	**RR per 10 dB increase in road traffic noise level (L_DEN_)**		1.05 (95% CI: 1.02–1.08) per 10 dB *	
	**Number of participants (^#^ evaluated studies)**		154,398 (26)	
	**Number of cases**		18,957	
			**Rating**	**Adjustment to rating**
**Quality assessment**	**Starting rating**		26 cross-sectional studies ^a^	2 (low)
	**Factors decreasing confidence**	**Risk of bias**	Serious ^b^	Downgrading
		**Inconsistency**	Serious ^c^	Downgrading
		**Indirectness**	None ^d^	No downgrading
		**Imprecision**	None ^e^	No downgrading
		**Publication bias**	Small probability of publication bias ^f^	Downgrading
	**Factors increasing confidence**	**Strength of association**	Small ^g^	No upgrading
		**Exposure-response gradient**	Evidence of an exposure-response gradient ^g^	Upgrading
		**Possible confounding**	No serious bias ^h^	Upgrading
	**Overall judgement of quality of evidence**			1 (very low)

* The estimate was based on 47 effect estimates; ^a^ Since only cross-sectional studies were available, we started with a grading of “low” (2); ^b^ In 12 studies, the participants were randomly selected taking into account exposure to road traffic noise; although the participants of these studies were randomly selected, 14 studies were originally not designed to investigate the impact of road traffic noise exposure; In 2 studies it was likely that participants were aware of the fact that they participated in a study investigating the impact of noise. In 8 studies, the participation rate was below 60%; for 16 studies, the participation rate was larger than 60%; ^c^ Results across studies differed in magnitude and direction of effect estimates (see Figure 4.3 of the complete review). This was confirmed by the results of the heterogeneity analyses, demonstrating “moderate” heterogeneity (I^2^_residual_ = 52.4%); ^d^ The evaluated studies assessed population, exposure, and outcome of interest; ^e^ We considered the results to be precise: the number of participants and the number of cases was large enough, and the 95% CI was sufficiently narrow; ^f^ There was reason to believe that there is some publication bias or small study bias (result of the Egger test provided evidence for small-study effects) (see also Figure 4.4 of the complete review); ^g^ Most studies found that the risk of hypertension increased when road traffic noise level increased (RR per 10 dB > 1). There was evidence of a significant exposure-response gradient: After aggregating the results of the evaluated studies, we found a significant effect size of 1.05 per 10 dB. The noise range of the studies under evaluation was 20–85 dB. This means that if road traffic noise level increases from 20 to 85 dB, the RR = 1.34. We found indications for an effect of exposure duration: The effect estimates turned out to be larger for the sample that lived for a longer period in the same house; ^h^ We did not find evidence to suggest that possible residual confounders or biases would reduce our effect estimate.

**Table A16:** Summary of findings table for the association between rail traffic noise exposure and the prevalence of hypertension.

Question	Does Exposure to Rail Traffic Noise Increase the Risk Of Hypertension			
**People**	Adult population (men and women)			
**Setting**	Residential setting: people living in several cities in Europe			
**Outcome**	The prevalence of hypertension			
**Summary of findings**	**RR per 10 dB increase in rail traffic noise level (L_DEN_)**		1.05 (95% CI: 0.88–1.26) per 10 dB	
	**Number of participants (^#^ evaluated studies)**		15,850 (5)	
	**Number of cases**		2059	
			**Rating**	**Adjustment to rating**
**Quality assessment**		**Starting rating**	5 cross-sectional studies ^#^	2 (low)
	**Factors decreasing confidence**	**Risk of bias**	Serious ^a^	Downgrading
		**Inconsistency**	Serious ^b^	Downgrading
		**Indirectness**	None ^c^	No downgrading
		**Imprecision**	None ^d^	No downgrading
		**Publication bias**	NA ^e^	No downgrading
	**Factors increasing confidence**	**Strength of association**	Small ^f^	No upgrading
		**Exposure-response gradient**	Evidence of a non-significant exposure-response gradient ^f^	No upgrading
		**Possible confounding**	No conclusions can be drawn ^g^	No upgrading
	**Overall judgement of quality of evidence**			0 (Very low)

^#^ Since only cross-sectional studies were available, we started with a grading of “low”(2); ^a^ In three studies, the participants were randomly selected taking into account road- and/or rail traffic noise exposure; although the participants of these studies were randomly selected, two other studies were originally not designed to investigate the impact of (rail) traffic noise exposure; In one study there is a chance that the participants were aware that they took part in a study investigating the impact of noise; in two other studies it is not very likely that participants were aware that they took part in a study investigating the impact of noise, since they were not originally set up to investigate the impact of noise. For one study, it was unclear whether participants were aware of taking part in a noise study. In two studies, response rates were below 60%; ^b^ Results across studies differed in the magnitude and direction of effect estimates (see Figure 4.5 of the complete review). This was confirmed by the results of the heterogeneity analyses, demonstrating “moderate” heterogeneity (I^2^_residual_ = 57.6%); ^c^ The evaluated studies assessed population, exposure, and outcome of interest; ^d^ We considered the results to be precise: the number of cases was large enough, and the 95% CI was sufficiently narrow; ^e^ Due to the low number of available effect estimates it was not possible to test for publication bias or small study bias; ^f^ Most studies found that the risk of hypertension increased when rail traffic noise level increased (RR per 10 dB > 1). There was evidence of a non-significant exposure-response gradient: After aggregating the results of the evaluated studies, we found a non-significant effect size of 1.05 per 10 dB. The noise range of the studies under evaluation was 30–80 dB (L_DEN_). This means that if rail traffic noise level increases from 30 to 80 dB, the RR = 1.28; ^g^ We were not able to draw any conclusions whether possible residual confounders or biases would reduce our effect estimate.

**Table A17:** Summary of findings table for the association between exposure to wind turbines and the prevalence of hypertension.

Question	Does Exposure to Noise from Wind Turbines Increase the Risk of Hypertension			
**People**	Adult population (men and women)			
**Setting**	Residential setting: people in the neighbourhood of wind turbines in The Netherlands and Sweden			
**Outcome**	The prevalence of hypertension			
**Summary of findings**	**RR per 10 dB increase in wind turbine noise level (SPL)**		-	
	**Number of participants (^#^ evaluated studies)**		1830 (3)	
	**Number of cases**		NR	
			**Rating**	**Adjustment to rating**
**Quality assessment**		**Starting rating**	3 cross-sectional studies ^#^	2 (low)
	**Factors decreasing confidence**	**Risk of bias**	Very serious ^a^	Downgrading
		**Inconsistency**	None ^b^	No downgrading
		**Indirectness**	None ^c^	No downgrading
		**Imprecision**	Limited ^d^	Downgrading
		**Publication bias**	NA ^e^	No downgrading
	**Factors increasing confidence**	**Strength of association**	NA ^f^	No upgrading
		**Exposure-response gradient**	NA ^f^	No upgrading
		**Possible confounding**	Serious bias cannot be ruled out ^g^	No upgrading
	**Overall judgement of quality of evidence**			0 (very low)

^#^ Since only cross-sectional studies were available, we started with a grading of “low” (2); ^a^ Methods used to select the population: response rates were in two of the three studies below 60%. The participants were randomly selected taking into account the distance between their house and a wind turbine (park); hypertension was in all cases measured by means of a questionnaire; ^b^ Although results across studies differed in the magnitude of effect estimates (see Figure of the complete review 4.6), all studies found a positive association between exposure to wind turbine noise and the prevalence of hypertension; ^c^ The evaluated studies assessed population, exposure, and outcome of interest; ^d^ We considered the results to be imprecise: we assessed that the number of cases was less than 200, which is small. The 95% CIs of the separate studies contained values below 0.5 and above 2.0; ^e^ Due to the low number of available effect estimates it was not possible to test for publication bias or small study bias; ^f^ We decided not to carry out a meta-analysis; ^g^ Although we did not find evidence to suggest that possible residual confounders or biases would reduce our effect estimate, the studies were unable to adjust for important confounders.

**Table A18:** Summary of findings table for the association between aircraft noise exposure and the incidence of hypertension.

Question	Does Exposure to Aircraft Noise Increase the Risk of Hypertension			
**People**	Adult population (men and women, 35–56 years)			
**Setting**	Residential setting: people living around Stockholm Arlanda airport in Sweden			
**Outcome**	The incidence of hypertension			
**Summary of findings**	**RR per 10 dB increase in aircraft noise level (L_DEN_)**		1.00 (0.77–1.30) per 10 dB	
	**Number of participants (^#^ studies)**		4712 (1)	
	**Number of cases**		1346	
			**Rating**	**Adjustment to rating**
**Quality assessment**	**Starting rating**		1 cohort study ^#^	4 (high)
	**Factors decreasing confidence**	**Risk of bias**	Serious limitations ^a^	Downgrading
		**Inconsistency**	NA ^b^	No downgrading
		**Indirectness**	None ^c^	No downgrading
		**Imprecision**	None ^d^	No downgrading
		**Publication bias**	NA ^e^	No downgrading
	**Factors increasing confidence**	**Strength of association**	Small ^f^	No upgrading
		**Exposure-response gradient**	No evidence of an exposure-response gradient ^f^	Nu upgrading
		**Possible confounding**	Non-residual misclassification of disease	No upgrading
	**Overall judgement of quality of evidence**			2 (Low) ^g^

^#^ Since a cohort study was available, we started with a grading of “high” (4); ^a^ Participants were a (partly) random selection from people participating in the Stockholm Preventive Programm. Hypertension was ascertained by both a clinical examination and a questionnaire; although it was not possible to exactly assess the attrition rate, it was probably > 20%; ^b^ Since only one study was evaluated, this criterion was not applied; ^c^ The study assessed population, exposure, and outcome of interest; ^d^ We considered the results to be precise: the sample was sufficiently large, and the 95% CI was sufficiently narrow; ^e^ Since only one study was evaluated, we were not able to test for publication bias; ^f^ We found a non-significant effect size of 1.00 per 10 dB. The noise range of the evaluated study was 45–65 dB (L_DEN_); ^g^ The overall judgement of the quality of evidence was graded as “moderate” (3). Since only one study was available, we downgraded the overall level of evidence to “low” (2).

**Table A19:** Summary of findings table for the association between road traffic noise exposure and the incidence of hypertension.

Question	Does Exposure to Road Traffic Noise Increase the Risk of Hypertension			
**People**	Adult population (men and women, 50–64 years)			
**Setting**	Residential setting: people living in Aarhus or Copenhagen (Denmark)			
**Outcome**	The incidence of hypertension			
**Summary of findings**	**RR per 10 dB increase in road traffic noise level (L_DEN_)**		0.97 (0.90–1.05) per 10 dB	
	**Number of participants (^#^ studies)**		43,635 (1)	
	**Number of cases**		3145	
			**Rating**	**Adjustment to rating**
**Quality assessment**	**Starting rating**		1 cohort study ^#^	4 (high)
	**Factors decreasing confidence**	**Risk of bias**	Serious limitations ^a^	Downgrading
		**Inconsistency**	Na ^b^	No downgrading
		**Indirectness**	None ^c^	No downgrading
		**Imprecision**	None ^d^	No downgrading
		**Publication bias**	NA ^e^	No downgrading
	**Factors increasing confidence**	**Strength of association**	NA ^f^	No upgrading
		**Exposure-response gradient**	No evidence of exposure-response gradient ^f^	No upgrading
		**Possible confounding**	None	No upgrading
	**Overall judgement of quality of evidence**			2 (low) ^g^

^#^ Since a cohort study was available, we started with a grading of “high” (4); ^a^ Participants were people participating in the DCH cohort. For this cohort, people living in Aarhus or Copenhagen, aged 50-64 years, and who were cancer-free, were randomly selected and invited. Attrition rate was > 20% after three years of follow-up time. Hypertension was ascertained by a questionnaire; ^b^ Since only one study was evaluated, this criterion was not applied; ^c^ The study assessed population, exposure, and outcome of interest; ^d^ We considered the results to be precise: the sample was sufficiently large, and the 95% CI was sufficiently narrow; ^e^ Since only one study was evaluated, we were not able to test for publication bias; ^f^ We found a non-significant effect size of less than 1.00 per 10 dB; ^g^ The overall judgement of the quality of evidence was graded “moderate”(3). Since only one study was available, we downgraded the overall level of evidence to “low” (2).

**Table A20:** Summary of findings table for the association between rail traffic noise exposure and the incidence of hypertension.

Question	Does Exposure to Rail Traffic Noise Increase the Risk of Hypertension			
**People**	Adult population (men and women, 50–64 years)			
**Setting**	Residential setting: people living in Aarhus or Copenhagen (Denmark)			
**Outcome**	The incidence of hypertension			
**Summary of findings**	**RR per 10 dB increase in road traffic noise level (L_DEN_)**		0.96 (0.88–1.04) per 10 dB	
	**Number of participants (^#^ studies)**		7249 (1)	
	**Number of cases**		3145	
			**Rating**	**Adjustment to rating**
**Quality assessment**	**Starting rating**		1 cohort study ^#^	4 (high)
	**Factors decreasing confidence**	**Risk of bias**	Serious limitations ^a^	Downgrading
		**Inconsistency**	NA ^b^	No downgrading
		**Indirectness**	None ^c^	No downgrading
		**Imprecision**	None ^d^	No downgrading
		**Publication bias**	NA ^e^	No downgrading
	**Factors increasing confidence**	**Strength of association**	NA ^f^	No upgrading
		**Exposure-response gradient**	No evidence of an exposure-response gradient ^f^	No upgrading
		**Possible confounding**	None	No upgrading
	**Overall judgement of quality of evidence**			2 (low) ^g^

^#^ Since a cohort study was available, we started with a grading of “high” (4); ^a^ Participants were people participating in the DCH cohort. For this cohort, people living in Aarhus or Copenhagen, aged 50–64 years. and who were cancer-free, were randomly selected and invited. Attrition rate was > 20% after three years of follow-up time. Hypertension was ascertained by a questionnaire; ^b^ Since only one study was evaluated, this criterion was not applied; ^c^ The study assessed population, exposure, and outcome of interest; ^d^ We considered the results to be precise: the sample was sufficiently large, and the 95% CI was sufficiently narrow; ^e^ Since only one study was evaluated, we were not able to test for publication bias; ^f^ We found a non-significant effect size of less than 1.00 per 10 dB; ^g^ The overall judgement of the quality of evidence was graded as “moderate”(3). Since only one study was available, we downgraded the overall level of evidence to “low” (2).

## Appendix D. Summary of Findings Tables Dealing with Studies on the Impact of Noise on Ischaemic Heart Disease

This appendix presents the summary of findings tables dealing with the studies on the impact of noise on IHD. An extensive description and the reasoning behind these tables can be found in the complete review in Section 11.2.

**Table A21:** Summary of findings table for the association between aircraft noise exposure and the prevalence of ischaemic heart disease.

Question	Does Exposure to Aircraft Noise Increase the Risk of IHD			
**People**	Adult population (men and women)			
**Setting**	Residential setting: people living in cities located around airports in Europe			
**Outcome**	The prevalence of IHD			
**Summary of findings**	**RR per 10 dB increase in aircraft noise level (L_DEN_)**		1.07 (95% CI: 0.94–1.23)	
	**Number of participants (^#^ studies)**		14,098 (2)	
	**Number of cases**		340	
			**Rating**	**Adjustment to rating**
**Quality assessment**	**Starting rating**		2 cross-sectional studies ^#^	2 (low)
	**Factors decreasing confidence**	**Risk of bias**	Serious ^a^	Downgrading
		**Inconsistency**	None ^b^	No downgrading
		**Indirectness**	None ^c^	No downgrading
		**Imprecision**	None ^d^	No downgrading
		**Publication bias**	NA ^e^	No downgrading
	**Factors increasing confidence**	**Strength of association**	Small ^f^	No upgrading
		**Exposure-response gradient**	Evidence of a non- significant exposure-response gradient ^f^	No upgrading
		**Possible confounding**	No conclusions can be drawn ^g^	No upgrading
	**Overall judgement of quality of evidence**			1 (very low)

^#^ Since only cross-sectional studies were available, we started with a grading of “low” (2); ^a^ In both studies, the population was selected randomly. Response rates were in both studies below 60%. In the studies, IHD was ascertained by means of a questionnaire only; one of the studies was not able to adjust for smoking; ^b^ Although results across studies differed in the magnitude if effect estimates, both studies found a positive association between exposure to aircraft noise and the prevalence of IHD (see Figure 5.1 of the complete review); ^c^ The studies assessed population, exposure and outcome of interest; ^d^ We considered the results to be precise: the number of cases was large enough, and the 95% CI was sufficiently narrow; ^e^ Due to the low number of available effect estimates, it was not possible to test for publication bias or small study bias; ^f^ Both studies found that the risk of IHD increased when air traffic noise level increased (RR per 10 dB > 1).There was evidence of a non-significant exposure-response gradient: After aggregating the results of the evaluated studies, we found a non-significant effect size of 1.07 per 10 dB. The noise range of the studies under evaluation was 30–70 dB (L_DEN_); ^g^ We were not able to draw any conclusions whether possible residual confounders or biases would reduce our effect estimate.

**Table A22:** Summary of findings table for the association between road traffic noise exposure and the prevalence of ischaemic heart disease.

Question	Does Exposure to Road Traffic Noise Increase the Risk of IHD			
**People**	Adult population (men and women)			
**Setting**	Residential setting: people living several cities in Europe			
**Outcome**	The prevalence of IHD			
**Summary of findings**	**RR per 10 dB increase in road traffic noise level (L_DEN_)**		1.24 (95% CI: 1.08–1.42) per 10 dB	
	**Number of participants (^#^ studies)**		25,682 (8)	
	**Number of cases**		1614	
			**Rating**	**Adjustment to rating**
**Quality assessment**	**Starting rating**		8 cross-sectional studies ^#^	2 (low) ^#^
	**Factors decreasing confidence**	**Risk of bias**	Serious ^a^	Downgrading
		**Inconsistency**	Serious ^b^	Downgrading
		**Indirectness**	None ^c^	No downgrading
		**Imprecision**	Minor ^d^	No downgrading
		**Publication bias**	NA ^e^	No downgrading
	**Factors increasing confidence**	**Strength of association**	Large ^f^	Upgrading
		**Exposure-response gradient**	Evidence of an exposure-response gradient ^f^	Upgrading
		**Possible confounding**	Possible bias ^g^	No upgrading
	**Overall judgement of quality of evidence**			2 (low)

^#^ Since only cross-sectional studies were available, we started with a grading of “low” (2); ^a^ Methods used to select the population: In 6 studies, the participants were randomly selected taking into account road traffic noise exposure. The response rates were below 60%. In four of the eight studies. In three of the included studies, exposure was assessed by noise models incorporated in GIS. The noise models used were able to estimate the noise levels at individual level. In four of the studies, IHD was ascertained by means of a questionnaire only; ^b^ Results across studies differed only in the magnitude of effect estimates (see Figure 5.2 of the complete review). This was confirmed by the results of the heterogeneity analyses, indicating “moderate” heterogeneity (I^2^_residual_ = 51.4%); ^c^ The studies assessed population, exposure and outcome of interest; ^d^ We considered the results to be less precise: the number of cases was large enough, and although the 95% CI contained values > 1.25, we considered the sample size as sufficiently large; ^e^ Due to the low number of available effect estimates, it was not possible to test for publication bias or small study bias; ^f^ All studies found that the risk of IHD increased when road traffic noise level increased (RR per 10 dB > 1). There was evidence of a significant exposure-response gradient: After aggregating the results of the evaluated studies, we found a significant effect size of 1.24 per 10 dB. The noise range of the studies under evaluation was 30–80 dB. This means that if road traffic noise level increases from 30 to 80 dB, the RR = 2.93; ^g^ Adjustment for smoking and indicators of air pollution were found to be important sources of heterogeneity.

**Table A23:** Summary of findings table for the association between rail traffic noise exposure and the prevalence of ischaemic heart disease.

Question		Does Exposure to Rail Traffic Noise Increase the Risk of IHD			
**People**		Adult population (men and women)			
**Setting**		Residential setting: people living several cities in Europe			
**Outcome**		The prevalence of IHD			
**Summary of findings**		RR per 10 dB increase in road traffic noise level (L_DEN_)		1.18 (95% CI: 0.82–1.68) per 10 dB	
		Number of participants (^#^ studies)		13,241 (4)	
		Number of cases		283	
				**Rating**	**Adjustment to rating**
**Quality assessment**	**Starting rating**			4 cross-sectional studies	2 (low) ^#^
	**Factors decreasing confidence**		**Risk of bias**	Serious ^a^	Downgrading
			**Inconsistency**	Serious ^b^	Downgrading
			**Indirectness**	None ^c^	No downgrading
			**Imprecision**	Minor ^d^	No downgrading
			**Publication bias**	NA ^e^	No downgrading
	**Factors increasing confidence**		**Strength of association**	Large, but non-significant ^f^	No upgrading
			**Exposure-response gradient**	Evidence of a non-significant exposure-response gradient ^f^	No upgrading
			**Possible confounding**	No conclusions can be drawn ^g^	No upgrading
	**Overall judgement of quality of evidence**				0 (very low)

^#^ Since only cross-sectional studies were available, we started with a grading of “low” (2); ^a^ Response rates were in two of the four studies below 60%. In all studies, IHD was ascertained by means of a questionnaire only; ^b^ Results across studies differed in the magnitude and direction of effect estimates (see Figure 5.7 of the complete review). This was confirmed by the results of the heterogeneity analyses, indicating “moderate” heterogeneity (I^2^_residual_ = 57.4%); ^c^ The studies assessed population, exposure and outcome of interest; ^d^ We considered the results to be less precise: the 95% CI contained values > 1.25; however, we considered the sample size to be sufficiently large; ^e^ Due to the low number of available effect estimates, it was not possible to test for publication bias or small study bias; ^f^ Most studies found that the risk of IHD increased when rail traffic noise level increased (RR per 10 dB > 1). There was evidence of a non-significant exposure-response gradient: After aggregating the results of the evaluated studies, we found a non-significant effect size of 1.18 per 10 dB. The noise range of the studies under evaluation was 30–80 dB. This means that if rail traffic noise level increases from 30 to 80 dB, the RR = 2.29; ^g^ We were not able to draw any conclusions whether possible residual confounders or biases would reduce our effect estimate.

**Table A24:** Summary of findings table for the association between aircraft noise exposure and the incidence of ischaemic heart disease.

Question	Does Exposure to Aircraft Noise Increase the Risk of IHD			
**People**	Adult population (men and women)			
**Setting**	Residential setting: people living in cities located around airports in the UK and USA			
**Outcome**	The incidence (hospital admissions) of IHD			
**Summary of findings**	RR per 10 dB increase in aircraft noise level (L_DEN_)		1.09 (95% CI: 1.04–1.15)	
	Number of participants (^#^ studies)		9,619,082 (2)	
	Number of cases		158,977	
			**Rating**	**Adjustment to rating**
**Quality assessment**	**Starting rating**		2 ecological studies	1 (very low) ^#^
	**Factors decreasing confidence**	**Risk of bias**	Serious ^a^	Downgrading
		**Inconsistency**	Limited ^b^	No downgrading
		**Indirectness**	None ^c^	No downgrading
		**Imprecision**	None ^d^	No downgrading
		**Publication bias**	NA	No downgrading
	**Factors increasing confidence**	**Strength of association**	Small ^f^	No upgrading
		**Exposure-response gradient**	Evidence of a significant exposure-response gradient ^f^	Upgrading
		**Possible confounding**	No conclusions can be drawn ^g^	No upgrading
	**Overall judgement of quality of evidence**			1 (very low)

^#^ Since only ecological studies were available, we started with a grading of “very low” (1); ^a^ Both ecological studies worked with a purposeful sample; so randomization and response rate is not an issue. Studies were not able to adjust for important confounders at individual level. Studies were unable to apply individual exposure estimates; ^b^ Although results across studies differed in the magnitude of effect estimates, both found a positive association between exposure to aircraft noise and the incidence of IHD (see Figure 5.1 of the complete review). This was confirmed by the results of the heterogeneity analyses, indicating “low” heterogeneity (I^2^_residual_ = 48.4%); ^c^ The studies assessed population, exposure and outcome of interest; ^d^ We considered the results to be precise: the number of participants, as well as the number of cases were much larger than 200, and the 95% CI did not contain values below 0.75 or above 1.25; ^e^ Due to the low number of available effect estimates, it was not possible to test for publication bias or small study bias; ^f^ There was evidence of a significant exposure-response gradient: We found a significant effect size of 1.09 per 10 dB across a noise range of 45 to ~65 dB, this means that if the aircraft noise level increases from 45 to 65 dB, the RR = 1.19; ^g^ We were not able to draw any conclusions whether possible residual confounders or biases would reduce our effect estimate.

**Table A25:** Summary of findings table for the association between road traffic noise exposure and the incidence of ischaemic heart disease: ecological studies.

Question	Does Exposure to Road Traffic Noise Increase the Risk of IHD			
**People**	Adult population (men and women)			
**Setting**	Residential setting: people living in Kaunas (Lithuania)			
**Outcome**	The incidence of IHD			
**Summary of findings**	RR per 10 dB increase in road traffic noise level (L_DEN_)		1.12 (95% CI: 0.85–1.48) per 10 dB	
	Number of participants (^#^ studies)		262,830 (1)	
	Number of cases		418	
			**Rating**	**Adjustment to rating**
**Quality assessment**	**Starting rating**		1 ecological study	1 (very low) ^#^
	**Factors decreasing confidence**	**Risk of bias**	Serious ^a^	Downgrading
		**Inconsistency**	Na ^b^	No downgrading
		**Indirectness**	None ^c^	No downgrading
		**Imprecision**	None ^d^	No downgrading
		**Publication bias**	NA ^e^	Downgrading
	**Factors increasing confidence**	**Strength of association**	NA ^f^	No upgrading
		**Exposure-response gradient**	Evidence of non-significant exposure-response gradient ^f^	No upgrading
		**Possible confounding**	No conclusions can be drawn ^g^	No upgrading
	**Overall judgement of quality of evidence**			0 (very low) ^h^

^#^ Since only one ecological study was available, we started with a grading of “very low” (1); ^a^ Ecological studies worked with a purposeful sample; so randomization and response rate is not an issue. The study was not able to adjust for important confounders at individual level, and was unable to apply individual exposure estimates; ^b^ Only one study was evaluated, so inconsistency was not an issue; ^c^ The study assessed population, exposure and outcome of interest; ^d^ Although the 95% CI contained values above 1.25, we considered the results to be precise: the number of participants, as well as the number of cases were much larger than 200; ^e^ Due to the low number of available effect estimates, it was not possible to test for publication bias or small study bias. However, when combining this study with the other case-control and cohort studies that investigated the association between road traffic noise and the incidence of IHD, the number of estimates became large enough to test for publication bias. The funnel plot (Figure 5.6 of the complete review) was somewhat a-symmetric, but the Egger test provided only weak evidence for small-study effects; ^f^ There was evidence of a non-significant exposure-response gradient: We found a non-significant effect size of 1.12 per 10 dB across a noise range of 55–75 dB; ^g^ We were not able to draw any conclusions whether possible residual confounders or biases would reduce our effect estimate; ^h^ The overall judgement of the quality of the evidence was “very low”(0). Downgrading of the overall level of evidence, because only one study was available, made no sense.

**Table A26:** Summary of findings table for the association between road traffic noise exposure and the incidence of ischaemic heart disease: cohort and case-control studies.

Question	Does Exposure to Road Traffic Noise Increase the Risk of IHD			
**People**	Adult population (men and women)			
**Setting**	Residential setting: people living several cities in Europe			
**Outcome**	The incidence of IHD			
**Summary of findings**	RR per 10 dB increase in road traffic noise level (L_DEN_)		1.08 (95% CI: 1.01–1.15) per 10 dB	
	Number of participants (^#^ studies)		67,224 (7)	
	Number of cases		7033	
			**Rating**	**Adjustment to rating**
**Quality assessment**	**Starting rating**		3 cohort studies, 4 case-control studies	4 (high) ^#^
	**Factors decreasing confidence**	**Risk of bias**	Limited ^a^	No downgrading
		**Inconsistency**	Limited ^b^	No downgrading
		**Indirectness**	None ^c^	No downgrading
		**Imprecision**	None ^d^	No downgrading
		**Publication bias**	Small probability of publication bias ^e^	Downgrading
	**Factors increasing confidence**	**Strength of association**	Small ^f^	No upgrading
		**Exposure-response gradient**	Evidence of an exposure-response gradient ^f^	Upgrading
		**Possible confounding**	No conclusions can be drawn ^g^	No upgrading
	**Overall judgement of quality of evidence**			4 (high)

^#^ Since cohort and case-control studies were available, we started with a grading of “high” (4); ^a^ In all the studies, the participants were randomly selected. For six studies, the response rate was higher than 60%; in all the cohort studies, the loss to follow-up was less than 20%. Methods to assess exposure: In three of the included studies, exposure was assessed by noise models incorporated in GIS. The noise models used were able to estimate the noise levels at individual level. In three other studies, noise exposure assessment was based on noise measurements in the direct living area of the participant; ^b^ Results across studies differed only in the magnitude of effect estimates (see Figure 5.3 of the complete review). The results of the heterogeneity analyses demonstrated no clear evidence for heterogeneity; ^c^ The study assessed population, exposure and outcome of interest; ^d^ We considered the results as precise: The number of participants and cases were much larger than 200, and the 95% CI did not contain values below 0.75 or above 1.25; ^e^ Due to the low number of available effect estimates, it was not possible to test for publication bias or small study bias. However, when combining these studies with the ecological study that investigated the association between road traffic noise and the incidence of IHD, the number of estimates became large enough to test for publication bias. The funnel plot (Figure 5.6) was somewhat a-symmetric, but the Egger test provided only weak evidence for small-study effects; ^f^ Most studies found that the risk of IHD increased when road traffic noise level increased (RR per 10 dB > 1). There was evidence of a significant exposure-response gradient: After aggregating the results of the evaluated studies, we found a significant effect size of 1.08 per 10 dB. The noise range of the studies under evaluation was 40–80 dB. This means that if road traffic noise level increases from 40 to 80 dB, the RR = 1.36; ^g^ We were not able to draw any conclusions whether possible residual confounders or biases would reduce our effect estimate.

**Table A27:** Summary of findings table for the association between aircraft noise exposure and mortality due to ischaemic heart disease: ecological studies.

Question	Does Exposure to Aircraft Noise Increase the Risk of IHD			
**People**	Adult population (men and women)			
**Setting**	Residential setting: people living in cities located around airports in the UK and The Netherlands			
**Outcome**	Mortality due to IHD			
**Summary of findings**	RR per 10 dB increase in aircraft noise level (L_DEN_)		1.04 (95% CI: 0.97–1.12)	
	Number of participants (^#^ studies)		3,897,645 (2)	
	Number of cases		26,066	
			**Rating**	**Adjustment to rating**
**Quality assessment**	**Starting rating**		2 ecological studies	1 (very low) ^#^
	**Factors decreasing confidence**	**Risk of bias**	Serious ^a^	Downgrading
		**Inconsistency**	Limited ^b^	No downgrading
		**Indirectness**	None ^c^	No downgrading
		**Imprecision**	None ^d^	No downgrading
		**Publication bias**	NA ^e^	No downgrading
	**Factors increasing confidence**	**Strength of association**	Small ^f^	No upgrading
		**Exposure-response gradient**	Evidence of a non-significant exposure-response gradient ^f^	No upgrading
		**Possible confounding**	No conclusions can be drawn ^g^	No upgrading
	**Overall judgement of quality of evidence**			0 (very low)

^#^ Since only ecological studies were available, we started with a grading of “very low” (0); ^a^ Both ecological studies worked with a purposeful sample; so randomization and response rate is not an issue. Studies were not able to adjust for important confounders at individual level. Studies were unable to apply individual exposure estimates; ^b^ Results across studies differed in the magnitude and direction of effect estimates (see Figure 5.1 of the complete review). This was not confirmed by the results of the heterogeneity analyses, demonstrating “low” heterogeneity (I^2^_residual_ = 39.7%); ^c^ The studies assessed population, exposure and outcome of interest; ^d^ We considered the results to be precise: Both the number of participants and cases were much larger than 200; the 95% CI did not contain values below 0.75 or above 1.25; ^e^ Due to the low number of available effect estimates, it was not possible to test for publication bias or small study bias; ^f^ One of the two studies found that the risk of IHD increased when air traffic noise level increased (RR per 10 dB > 1). There was evidence of a non-significant exposure-response gradient: After aggregating the results of the evaluated studies, we found a non-significant effect size of 1.04 per 10 dB. The noise range of the studies under evaluation was 40–65 dB; ^g^ We were not able to draw any conclusions whether possible residual confounders or biases would reduce our effect estimate.

**Table A28:** Summary of findings table for the association between aircraft noise exposure and mortality due to ischaemic heart disease: cohort studies.

Question	Does Exposure to Aircraft Noise Increase the Risk of IHD			
**People**	Adult population (men and women)			
**Setting**	Residential setting: people living in Switzerland			
**Outcome**	Mortality due to IHD			
**Summary of findings**	RR per 10 dB increase in aircraft noise level (L_DEN_)		1.04 (95% CI: 0.98–1.11) per 10 dB	
	Number of participants (^#^ studies)		4,580,311 (1)	
	Number of cases		15,532	
			**Rating**	**Adjustment to rating**
**Quality assessment**	**Starting rating**		1 cohort study	4 (high) ^#^
	**Factors decreasing confidence**	**Risk of bias**	Serious ^a^	Downgrading
		**Inconsistency**	Na ^b^	No downgrading
		**Indirectness**	None ^c^	No downgrading
		**Imprecision**	None ^d^	No downgrading
		**Publication bias**	NA ^e^	No downgrading
	**Factors increasing confidence**	**Strength of association**	Small ^f^	No upgrading
		**Exposure-response gradient**	Evidence of a non-significant exposure-response gradient ^f^	No upgrading
		**Possible confounding**	No conclusions can be drawn ^g^	No upgrading
	**Overall judgement of quality of evidence**			2 (low) ^h^

^#^ Since a cohort study was available, we started with a grading of “high” (4); ^a^ Aircraft noise levels were available at 100 × 100 m grids and the study suffered from a lack of information about important life style factors; ^b^ Only one study was evaluated, so inconsistency was not an issue (see Figure 5.1 of the complete review); ^c^ The study assessed population, exposure and outcome of interest. ^d^ We considered the results to be precise: Both the number of participants and cases were much larger than 200. The 95% CI did not contain values below 0.75 or above 1.25; ^e^ Due to the low number of available effect estimates, it was not possible to test for publication bias or small study bias; ^f^ There was evidence of a non-significant exposure-response gradient: We found a non-significant effect size of 1.04 per 10 dB across a noise range of 40 to 60 dB; ^g^ We were not able to draw any conclusions whether possible residual confounders or biases would reduce our effect estimate; ^h^ We graded the overall quality of evidence as “moderate”. Since only one study was available, we downgraded the overall level of evidence to “low” (2).

**Table A29:** Summary of findings table for the association between road traffic noise exposure and mortality due to ischaemic heart disease: cohort and case-control studies.

Question	Does Exposure to Road Traffic Noise Increase the Risk of IHD			
**People**	Adult population (men and women)			
**Setting**	Residential setting: people living several cities in Europe			
**Outcome**	Mortality due to IHD			
**Summary of findings**	RR per 10 dB increase in road traffic noise level (L_DEN_)		1.05 (95% CI: 0.97–1.13) per 10 dB	
	Number of participants (^#^ studies)		532,268 (3)	
	Number of cases		6884	
			**Rating**	**Adjustment to rating**
**Quality assessment**	**Starting rating**		1 cohort studies, 2 case-control studies	4 (high) ^#^
	**Factors decreasing confidence**	**Risk of bias**	Limited ^a^	Downgrading
		**Inconsistency**	Limited ^b^	No downgrading
		**Indirectness**	None ^c^	No downgrading
		**Imprecision**	None ^d^	No downgrading
		**Publication bias**	NA ^e^	No downgrading
	**Factors increasing confidence**	**Strength of association**	Small ^f^	No upgrading
		**Exposure-response gradient**	Evidence of a non-significant exposure-response gradient ^f^	No upgrading
		**Possible confounding**	No conclusions can be drawn ^g^	No upgrading
	**Overall judgement of quality of evidence**			3 (moderate)

^#^ Since cohort and case-control studies were available, we started with a grading of “high” (4); ^a^ For the largest of the three studies, there was a possible risk of bias since there were worries with regard to exposure assessment, and one was not able to adjust for smoking; ^b^ Results across studies differed in the magnitude and direction of effect estimates (see Figure 5.5 of the complete review). This was not confirmed by the heterogeneity analyses, demonstrating “low” heterogeneity (I^2^_residual_ = 34.9%); ^c^ The study assessed population, exposure and outcome of interest; ^d^ We considered the results to be precise: Both the number of participants and cases were much larger than 200. The 95% CI did not contain values below 0.75 or above 1.25; ^e^ Due to the low number of available effect estimates, it was not possible to test for publication bias or small study bias; ^f^ Most studies found that the risk of IHD increased when road traffic noise level increased (RR per 10 dB > 1). There was evidence of a non-significant exposure-response gradient: After aggregating the results of the evaluated studies, we found a non-significant effect size of 1.05 per 10 dB. The noise range of the studies under evaluation was 42–70 dB; ^g^ We were not able to draw any conclusions whether possible residual confounders or biases would reduce our effect estimate.

## Appendix E. Summary of Findings Tables Dealing with Studies on the Impact of Noise on Stroke

This appendix presents the summary of findings tables dealing with the studies on the impact of noise on stroke. An extensive description and the reasoning behind these tables can be found in the complete review in Section 11.3.

**Table A30:** Summary of findings table for the association between aircraft noise exposure and the prevalence of stroke.

Question	Does Exposure to Aircraft Noise Increase the Risk of Stroke			
**People**	Adult population (men and women)			
**Setting**	Residential setting: people living in cities located around airports in Europe and The Netherlands			
**Outcome**	The prevalence of stroke			
**Summary of findings**	RR per 10 dB increase in aircraft noise level (L_DEN_)		1.02 (95% CI: 0.80–1.28)	
	Number of participants (^#^ studies)		14,098 (2)	
	Number of cases		151	
			**Rating**	**Adjustment to rating**
**Quality assessment**	**Starting rating**		2 cross-sectional studies ^#^	2 (low)
	**Factors decreasing confidence**	**Risk of bias**	Serious ^a^	Downgrading
		**Inconsistency**	Limited ^b^	No downgrading
		**Indirectness**	None ^c^	No downgrading
		**Imprecision**	Serious ^d^	Downgrading
		**Publication bias**	NA ^e^	No downgrading
	**Factors increasing confidence**	**Strength of association**	Small ^f^	No upgrading
		**Exposure-response gradient**	Evidence of a non- significant exposure-response gradient ^f^	No upgrading
		**Possible confounding**	No conclusions can be drawn ^g^	No upgrading
	**Overall judgement of quality of evidence**			0 (very low)

^#^ Since only cross-sectional studies were available, we started with a grading of “low” (2); ^a^ Response rates were in both studies below 60%. In the studies, stroke was ascertained by means of a questionnaire only; one of the two studies was not able to adjust for smoking; ^b^ Results between studies differed in the magnitude and direction of effect estimates (see Figure 6.1 of the complete review). This was not confirmed by the result of the heterogeneity analysis, demonstrating “low” heterogeneity (I^2^_residual_ = 0.0%); ^c^ The studies assessed population, exposure and outcome of interest; ^d^ We considered the results to be imprecise: The number of cases was smaller than 200, and the 95% CI was judged as not sufficiently narrow; ^e^ Due to the low number of available effect estimates, it was not possible to test for publication bias or small study bias; ^f^ One the two studies found that the risk of stroke increased when air traffic noise level increased (RR per 10 dB > 1). There was evidence of a non-significant exposure-response gradient: After aggregating the results of the evaluated studies, we found a non-significant effect size of 1.02 per 10 dB. The noise range of the studies under evaluation was 30–75 dB; ^g^ We were not able to draw any conclusions whether possible residual confounders or biases would reduce our effect estimate.

**Table A31:** Summary of findings table for the association between road traffic noise exposure and the prevalence of stroke.

Question	Does Exposure to Road Traffic Noise Increase the Risk of Stroke			
**People**	Adult population (men and women)			
**Setting**	Residential setting: people living in cities located around airports in Europe and The Netherlands			
**Outcome**	The prevalence of stroke			
**Summary of findings**	RR per 10 dB increase in road traffic noise level (L_DEN_)		1.00 (95% CI: 0.91–1.10) per 10 dB	
	Number of participants (^#^ studies)		14,098 (2)	
	Number of cases		151	
			**Rating**	**Adjustment to rating**
**Quality assessment**	**Starting rating**		2 cross-sectional studies ^#^	2 (low)
	**Factors decreasing confidence**	**Risk of bias**	Serious ^a^	Downgrading
		**Inconsistency**	Limited ^b^	No downgrading
		**Indirectness**	None ^c^	No downgrading
		**Imprecision**	Serious ^d^	Downgrading
		**Publication bias**	NA ^e^	No downgrading
	**Factors increasing confidence**	**Strength of association**	NA ^f^	No upgrading
		**Exposure-response gradient**	No evidence of an exposure-response gradient ^f^	No upgrading
		**Possible confounding**	No conclusions can be drawn ^g^	No upgrading
	**Overall judgement of quality of evidence**			0 (very low)

^#^ Since only cross-sectional studies were available, we started with a grading of “low” (2); ^a^ Response rates were in both studies below 60%. In the studies, stroke was ascertained by means of a questionnaire only; one of the two studies was not able to adjust for smoking; ^b^ Results between studies differed in the magnitude and direction of effect estimates (see Figure 6.2 of the complete review). This was not confirmed by the result of the heterogeneity analysis, demonstrating “low” heterogeneity (I^2^_residual_ = 0.0%); ^c^ The studies assessed population, exposure and outcome of interest; ^d^ We considered the results to be imprecise since the number of cases was smaller than 200. The 95% CI was judged as sufficiently narrow; ^e^ Due to the low number of available effect estimates, it was not possible to test for publication bias or small study bias; ^f^ One the two studies found that the risk of stroke increased when road traffic noise level increased (RR per 10 dB > 1). There was no evidence of an exposure-response gradient: After aggregating the results of the evaluated studies, we found a non-significant effect size of 1.00 per 10 dB. The noise range of the studies under evaluation was 30–75 dB; ^g^ We were not able to draw any conclusions whether possible residual confounders or biases would reduce our effect estimate.

**Table A32:** Summary of findings table for the association between rail traffic noise exposure and the prevalence of stroke.

Question	Does Exposure to Rail Traffic Noise Increase the Risk of Stroke			
**People**	Adult population (men and women)			
**Setting**	Residential setting: people living in cities around airports in The Netherlands			
**Outcome**	The prevalence of stroke			
**Summary of findings**	RR per 10 dB increase in road traffic noise level (L_DEN_)		1.07 (95% CI: 0.92–1.25) per 10 dB	
	Number of participants (^#^ studies)		9365 (1)	
	Number of cases		89	
			**Rating**	**Adjustment to rating**
**Quality assessment**	**Starting rating**		1 cross-sectional study ^#^	2 (low)
	**Factors decreasing confidence**	**Risk of bias**	Serious ^a^	Downgrading
		**Inconsistency**	NA ^b^	No downgrading
		**Indirectness**	None ^c^	No downgrading
		**Imprecision**	Serious ^d^	Downgrading
		**Publication bias**	NA ^e^	No downgrading
	**Factors increasing confidence**	**Strength of association**	Small, but non-significant ^f^	No upgrading
		**Exposure-response gradient**	Evidence of a non-significant exposure-response gradient ^f^	No upgrading
		**Possible confounding**	No conclusions can be drawn ^g^	No upgrading
	**Overall judgement of quality of evidence**			0 (very low) ^h^

^#^ Since one cross-sectional study was available, we started with a grading of “low” (2); ^a^ Response rate was below 60%, and stroke was ascertained by means of a questionnaire only; ^b^ NA; ^c^ The study assessed population, exposure and outcome of interest; ^d^ We considered the results to be imprecise: Although the 95% CI was considered as sufficiently narrow, we considered the number of cases to be small; ^e^ Due to the low number of available effect estimates, it was not possible to test for publication bias or small study bias; ^f^ The evaluated study found that the risk of stroke increased when rail traffic noise level increased (RR per 10 dB > 1). There was evidence of a non-significant exposure-response gradient: We found a non-significant effect size of 1.07 per 10 dB. The noise range of the study under evaluation was 30–65 dB; ^g^ We were not able to draw any conclusions whether possible residual confounders or biases would reduce our effect estimate; ^h^ We graded the overall quality of the evidence to be “very low” (0). Grading the overall judgement of the quality of evidence down with one level was not considered to be useful. Despite the fact that only one study was available, we did not downgrade the overall level of evidence. The overall judgement of the quality of evidence was already judged as “very low”.

**Table A33:** Summary of findings table for the association between aircraft noise exposure and the incidence of stroke: ecological studies.

Question	Does Exposure to Aircraft Noise Increase the Risk of Stroke			
**People**	Adult population (men and women)			
**Setting**	Residential setting: people living in cities located around airports in the UK and USA			
**Outcome**	The incidence (hospital admissions) of stroke			
**Summary of findings**	RR per 10 dB increase in aircraft noise level (L_DEN_)		1.05 (95% CI: 0.96–1.15)	
	Number of participants (^#^ studies)		9,619,082 (2)	
	Number of cases		97,949	
			**Rating**	**Adjustment to rating**
**Quality assessment**	**Starting rating**		2 ecological studies	1 (very low)
	**Factors decreasing confidence**	**Risk of bias**	Serious ^a^	Downgrading
		**Inconsistency**	Serious ^b^	Downgrading
		**Indirectness**	None ^c^	No downgrading
		**Imprecision**	None ^d^	No downgrading
		**Publication bias**	NA	No downgrading
	**Factors increasing confidence**	**Strength of association**	Small ^f^	No upgrading
		**Exposure-response gradient**	Evidence of a non-significant exposure-response gradient ^f^	No upgrading
		**Possible confounding**	No conclusions can be drawn ^g^	No upgrading
	**Overall judgement of quality of evidence**			0 (very low)

^#^ Since only ecological studies were available, we started with a grading of “very low” (1); ^a^ Both ecological studies worked with a purposeful sample; so randomization and response rate is not an issue. Studies were not able to adjust for important confounders at individual level. Studies were unable to apply individual exposure estimates; ^b^ Results between studies differed in the magnitude and direction of effect estimates (see Figure 6.1 of the complete review). This was confirmed by the result of the heterogeneity analysis, indicating “strong” heterogeneity (I^2^_residual_ = 82.7%); ^c^ The studies assessed population, exposure and outcome of interest; ^d^ We considered the results to be precise: Both the number of participants and cases were much larger than 200. The 95% CI did not contain values below 0.75 or above 1.25; ^e^ Due to the low number of available effect estimates, it was not possible to test for publication bias or small study bias; ^f^ One the two studies found that the risk of stroke increased when air traffic noise level increased (RR per 10 dB > 1). There was evidence of a non-significant exposure-response gradient: After aggregating the results of the evaluated studies, we found a non-significant effect size of 1.05 per 10 dB. The noise range of the studies under evaluation was 40 to approximately 65 dB; ^g^ We were not able to draw any conclusions whether possible residual confounders or biases would reduce our effect estimate.

**Table A34:** Summary of findings table for the association between road traffic noise exposure and the incidence of stroke.

Question	Does Exposure to Road Traffic Noise Increase the Risk of Stroke			
**People**	Adult population (men and women)			
**Setting**	Residential setting: people living in several cities in Denmark			
**Outcome**	The incidence of stroke			
**Summary of findings**	RR per 10 dB increase in road traffic noise level (L_DEN_)		1.14 (95% CI: 1.03–1.25) per 10 dB	
	Number of participants (^#^ studies)		51,485 (1)	
	Number of cases		1881	
			**Rating**	**Adjustment to Rating**
**Quality assessment**	**Starting rating**		1 cohort study	4 (high)
	**Factors decreasing confidence**	**Risk of bias**	Limited ^a^	No downgrading
		**Inconsistency**	NA ^b^	No downgrading
		**Indirectness**	None ^c^	No downgrading
		**Imprecision**	None ^d^	No downgrading
		**Publication bias**	NA ^e^	No downgrading
	**Factors increasing confidence**	**Strength of association**	Small ^f^	No upgrading
		**Exposure-response gradient**	Evidence of an exposure-response gradient ^f^	Upgrading
		**Possible confounding**	No conclusions can be drawn ^g^	No upgrading
	**Overall judgement of quality of evidence**			3 (moderate) ^h^

^#^ Since one cohort study was available, we started with a grading of ”high” (4); ^a^ No limitations in study design found; ^b^ Only one study was evaluated, so inconsistency was not an issue; ^c^ The study assessed population, exposure and outcome of interest; ^d^ We considered the results of the study to be precise: Both the number of participants and cases were much larger than 200. The 95% CI did not contain values below 0.75 or above 1.25; ^e^ The number of available effect estimates was too small to test for publication bias; ^f^ The evaluated study found that the risk of stroke increased when road traffic noise level increased (RR per 10 dB > 1). There was evidence of a significant exposure-response gradient: We found a significant effect size of 1.14 per 10 dB. The noise range of the study under evaluation was approximately 50 to 70 dB. This means that if the road traffic noise level increases from 50 to 70 dB, the RR = 1.30; ^g^ We were not able to draw any conclusions whether possible residual confounders or biases would reduce our effect estimate; ^h^ We graded the overall quality of the evidence to be “high” (4). Since only one study was available, we downgraded the overall level of evidence to “moderate” (3).

**Table A35:** Summary of findings table for the association between aircraft noise exposure and mortality due to stroke: ecological studies.

Question	Does Exposure to Aircraft Noise Increase the Risk of Stroke			
**People**	Adult population (men and women)			
**Setting**	Residential setting: people living in cities located around airports in the UK and The Netherlands			
**Outcome**	Mortality due to stroke			
**Summary of findings**	RR per 10 dB increase in aircraft noise level (L_DEN_)		1.07 (95% CI: 0.98–1.17)	
	Number of participants (^#^ studies)		3,897,645 (2)	
	Number of cases		12,086	
			**Rating**	**Adjustment to rating**
**Quality assessment**	**Starting rating**		2 ecological studies	1 (very low)
	**Factors decreasing confidence**	**Risk of bias**	Serious ^a^	Downgrading
		**Inconsistency**	Limited ^b^	No downgrading
		**Indirectness**	None ^c^	No downgrading
		**Imprecision**	None ^d^	No downgrading
		**Publication bias**	NA	No downgrading
	**Factors increasing confidence**	**Strength of association**	Small ^f^	No upgrading
		**Exposure-response gradient**	Evidence of a non- significant exposure-response gradient ^f^	No upgrading
		**Possible confounding**	No conclusions can be drawn ^g^	No upgrading
	**Overall judgement of quality of evidence**			0 (very low)

^#^ Since we only ecological studies were available, we started with a grading of “very low” (1); ^a^ Both ecological studies worked with a purposeful sample; so randomization and response rate is not an issue. Studies were not able to adjust for important confounders at individual level. Studies were unable to apply individual exposure estimates; ^b^ Results between studies differed in the magnitude of effect estimates (see Figure 6.1 of the complete review). The result of the heterogeneity analysis demonstrated “low” heterogeneity (I^2^_residual_ = 28.5%); ^c^ The studies assessed population, exposure and outcome of interest; ^d^ We considered the results to be precise: Both the number of participants and cases were much larger than 200. The 95% CI did not contain values below 0.75 or above 1.25; ^e^ Due to the low number of available effect estimates, it was not possible to test for publication bias or small study bias; ^f^ Both studies found that the risk of stroke increased when air traffic noise level increased (RR per 10 dB > 1). There was evidence of a non-significant exposure-response gradient: After aggregating the results of the evaluated studies, we found a non-significant effect size of 1.07 per 10 dB. The noise range of the studies under evaluation was approximately 40 to 65 dB. This means that if the aircraft noise level increases from 40 to 65 dB, the RR = 1.18; ^g^ We were not able to draw any conclusions whether possible residual confounders or biases would reduce our effect estimate.

**Table A36:** Summary of findings table for the association between aircraft noise exposure and the mortality due to stroke: cohort studies.

Question	Does Exposure to Air Traffic Noise Increase the Risk of Stroke			
**People**	Adult population (men and women)			
**Setting**	Residential setting: people living in several cities near airports in Switzerland			
**Outcome**	Mortality due to stroke			
**Summary of findings**	RR per 10 dB increase in air traffic noise level (L_DEN_)		0.99 (95% CI: 0.94–1.04) per 10 dB	
	Number of participants (^#^ studies)		4,580,311 (1)	
	Number of cases		25,231	
			**Rating**	**Adjustment to rating**
**Quality assessment**	**Starting rating**		1 cohort study	4 (high)
	**Factors decreasing confidence**	**Risk of bias**	Limited ^a^	No downgrading
		**Inconsistency**	NA ^b^	No downgrading
		**Indirectness**	None ^c^	No downgrading
		**Imprecision**	None ^d^	No downgrading
		**Publication bias**	NA ^e^	No downgrading
	**Factors increasing confidence**	**Strength of association**	NA ^f^	No upgrading
		**Exposure-response gradient**	No evidence of an exposure-response gradient ^f^	No upgrading
		**Possible confounding**	No conclusions can be drawn ^g^	No upgrading
	**Overall judgement of quality of evidence**			3 (moderate) ^g^

^#^ Since one cohort study was available, we started with a grading of “high” (4); ^a^ No limitations in study design found; ^b^ Only one study was evaluated, so inconsistency was not an issue; ^c^ The study assessed population, exposure and outcome of interest; ^d^ We considered the results to be precise: Both the number of participants and cases were much larger than 200. The 95% CI did not contain values below 0.75 or above 1.25; ^e^ The number of available effect estimates was too small to test for publication bias; ^f^ The evaluated study did not find that the risk of stroke increased when air traffic noise level increased (RR per 10 dB < 1). There was no evidence of a gradient: We found a non-significant effect size of 0.99 per 10 dB. The noise range of the study under evaluation was approximately 40 to 65 dB; ^g^ We graded the overall quality of the evidence to be “high”. Since only one study was available, we downgraded the overall level of evidence “moderate” (3).

**Table A37:** Summary of findings table for the association between road traffic noise exposure and mortality due to stroke.

Question	Does Exposure to Road Traffic Noise Increase the Risk of Stroke			
**People**	Adult population (men and women)			
**Setting**	Residential setting: people living in several cities in Denmark, The Netherlands and Canada			
**Outcome**	Mortality due to stroke			
**Summary of findings**	RR per 10 dB increase in road traffic noise level (L_DEN_)		0.87 (95% CI: 0.71–1.06) per 10 dB	
	Number of participants (^#^ studies)		581,517 (3)	
	Number of cases		2634	
			**Rating**	**Adjustment to Rating**
**Quality assessment**	**Starting rating**		3 cohort studies	4 (high)
	**Factors decreasing confidence**	**Risk of bias**	Limited ^a^	No downgrading
		**Inconsistency**	Serious ^b^	Downgrading
		**Indirectness**	None ^c^	No downgrading
		**Imprecision**	Limited ^d^	No downgrading
		**Publication bias**	NA ^e^	No downgrading
	**Factors increasing confidence**	**Strength of association**	NA ^f^	No upgrading
		**Exposure-response gradient**	No evidence of an exposure-response gradient ^f^	No upgrading
		**Possible confounding**	No conclusions can be drawn ^g^	No upgrading
	**Overall judgement of quality of evidence**			3 (moderate)

^#^ Since cohort studies were available, we started with a grading of “high” (4); ^a^ No limitations in study design found; ^b^ Results across studies differed in the magnitude and direction of effect estimates (see also Figure 6.2). This was confirmed by the results of the heterogeneity analysis, demonstrating “strong” heterogeneity (I^2^_residual_ = 78.0%); ^c^ The study assessed population, exposure and outcome of interest; ^d^ We considered the results to be precise enough: Both the number of participants and cases were much larger than 200. However, the 95% CI did contain values below 0.75; ^e^ The number of available effect estimates were too small to test for publication bias; ^f^ Only one of the evaluated studies found that the risk of stroke increased when road traffic noise level increased (RR per 10 dB > 1). There was no evidence of an exposure-response gradient: After aggregating the results of the studies, a non-significant effect size of 0.87 per 10 dB across a noise range of ~50 to 70 dB was found; ^g^ We were not able to draw any conclusions whether possible residual confounders or biases would reduce our effect estimate.

## Appendix F. Summary of Findings Tables Dealing with Studies on the Impact of Noise on Diabetes

This appendix presents the summary of findings tables dealing with the studies on the impact of noise on diabetes. An extensive description and the reasoning behind these tables can be found in the complete review in Section 11.4.

**Table A38:** Summary of findings table for the association between aircraft noise exposure and the prevalence of diabetes.

Question	Does Exposure to Aircraft Noise Increase the Risk of Diabetes			
**People**	Adult population (men and women)			
**Setting**	Residential setting: people living in cities located around airports in The Netherlands			
**Outcome**	The prevalence of diabetes			
**Summary of findings**	RR per 10 dB increase in aircraft noise level (L_DEN_)		1.01 (95% CI: 0.78–1.31)	
	Number of participants (^#^ studies)		9365 (1)	
	Number of cases		89	
			**Rating**	**Adjustment to Rating**
**Quality assessment**	**Starting rating**		1 cross-sectional study ^#^	2 (low)
	**Factors decreasing confidence**	Risk of bias	Serious ^a^	Downgrading
		Inconsistency	NA ^b^	No downgrading
		Indirectness	None ^c^	No downgrading
		Imprecision	Serious ^d^	Downgrading
		Publication bias	NA ^e^	No downgrading
	**Factors increasing confidence**	Strength of association	Small ^f^	No upgrading
		Exposure-response gradient	Evidence of a non- significant exposure-response gradient ^f^	No upgrading
		Possible confounding	No conclusions can be drawn ^g^	No upgrading
	**Overall judgement of quality of evidence**			0 (very low) ^h^

^#^ Since only one cross-sectional study was available, we started with a grading of “low” (2); ^a^ The response rates was below 60%. Diabetes was ascertained by means of a questionnaire only; the study was not able to adjust for smoking; ^b^ Since only one study is available, this criterion is not applicable; ^c^ The study assessed population, exposure and outcome of interest; ^d^ We considered the results to be imprecise: The number of cases was small, and the 95% CI was not sufficiently narrow; ^e^ Since the results of only one study were available it was not possible to test for publication bias or small study bias; ^f^ The evaluated study found that the risk of diabetes increased when air traffic noise level increased (RR per 10 dB > 1). There was evidence of a non-significant exposure-response gradient: we found a non-significant effect size of 1.01 per 10 dB. The noise range of the studies under evaluation was 30–65 dB. this means that if the air traffic noise level increases from 30 to 65 dB, the RR = 1.04; ^g^ We were not able to draw any conclusions whether possible residual confounders or biases would reduce our effect estimate; ^h^ We graded overall quality of the evidence to be “very low” (0). Despite the fact that only one study was available, it was not useful to downgrade the overall quality of evidence.

**Table A39:** Summary of findings table for the association between road traffic noise exposure and the prevalence of diabetes.

Question	Does Exposure to Road Traffic Noise Increase the Risk of Diabetes			
**People**	Adult population (men and women)			
**Setting**	Residential setting: people living in cities located around airports in The Netherlands and Stockholm			
**Outcome**	The prevalence of diabetes			
**Summary of findings**	RR per 10 dB increase in road noise level (L_DEN_)		NR	
	Number of participants (^#^ studies)		11,460 (2)	
	Number of cases		242	
			**Rating**	**Adjustment to Rating**
**Quality assessment**	**Starting rating**		2 cross-sectional study ^#^	2 (low)
	**Factors decreasing confidence**	Risk of bias	Serious ^a^	Downgrading
		Inconsistency	Limited ^b^	No downgrading
		Indirectness	None ^c^	No downgrading
		Imprecision	Serious ^d^	Downgrading
		Publication bias	NA ^e^	No downgrading
	**Factors increasing confidence**	Strength of association	NA ^f^	No upgrading
		Exposure-response gradient	NA ^f^	No upgrading
		Possible confounding	No conclusions can be drawn ^g^	No upgrading
	**Overall judgement of quality of evidence**			0 (very low)

^#^ Since only cross-sectional studies were available, we started with a grading of “low” (2); ^a^ In one of the studies, the response rate was below 60%. In the studies, diabetes was ascertained by means of a questionnaire only; ^b^ Results of the studies differed in the magnitude of effect estimates; ^c^ The studies assessed population, exposure and outcome of interest; ^d^ We considered the results of the studies to be imprecise: Although the number of cases was > 200, the 95% CIs of the separate studies were not sufficiently narrow; ^e^ Since the results of only two studies were available it was not possible to test for publication bias or small study bias; ^f^ Both studies found that the risk of diabetes increased when road traffic noise level increased (RR per 10 dB > 1). A meta-analysis was not carried out; ^g^ We were not able to draw any conclusions whether possible residual confounders or biases would reduce our effect estimate.

**Table A40:** Summary of findings table for the association between rail traffic noise exposure and the prevalence of diabetes.

Question	Does Exposure to Rail Traffic Noise Increase the Risk of Diabetes			
**People**	Adult population (men and women)			
**Setting**	Residential setting: people living in cities located around airports in The Netherlands			
**Outcome**	The prevalence of diabetes			
**Summary of findings**	RR per 10 dB increase in rail noise level (L_DEN_)		0.21 (95% CI: 0.05–0.82)	
	Number of participants (^#^ studies)		9365 (1)	
	Number of cases		89	
			**Rating**	**Adjustment to Rating**
**Quality assessment**	**Starting rating**		1 cross-sectional study ^#^	2 (low)
	**Factors decreasing confidence**	Risk of bias	Serious ^a^	Downgrading
		Inconsistency	NA ^b^	No downgrading
		Indirectness	None ^c^	No downgrading
		Imprecision	Serious ^d^	Downgrading
		Publication bias	NA ^e^	No downgrading
	**Factors increasing confidence**	Strength of association	Small ^f^	No upgrading
		Exposure-response gradient	NA ^f^	No upgrading
		Possible confounding	No conclusions can be drawn ^g^	No upgrading
	**Overall judgement of quality of evidence**			0 (very low) ^h^

^#^ Since only one cross-sectional study was available, we started with a grading of “low” (2); ^a^ The response rate was below 60%. Diabetes was ascertained by means of a questionnaire only; ^b^ Since only one study is available, this criterion is not applicable; ^c^ The study assessed population, exposure and outcome of interest; ^d^ We considered the results to be imprecise: The number of cases was small, and the 95% CI was not sufficiently narrow; ^e^ Since the results of only one study were available, it was not possible to test for publication bias or small study bias; ^f^ In the evaluated study a health promoting effect of noise was found; ^g^ We were not able to draw any conclusions whether possible residual confounders or biases would reduce our effect estimate; ^h^ We graded the overall quality of the evidence to be “very low”(0). Despite the fact that only one study was available, it was not useful to downgrade the overall quality of evidence.

**Table A41:** Summary of findings table for the association between exposure to noise from wind turbines and the prevalence of diabetes.

Question	Does Exposure to Noise from Wind Turbines Increase the Risk of Diabetes			
**People**	Adult population (men and women)			
**Setting**	Residential setting: people in the neighbourhood of wind turbines in The Netherlands and Sweden			
**Outcome**	The prevalence of diabetes			
**Summary of findings**	RR per 10 dB increase in wind turbine noise level (SPL)		-	
	Number of participants (^#^ evaluated studies)		1830 (3)	
	Number of cases		NR	
			**Rating**	**Adjustment to rating**
**Quality assessment**	**Starting rating**		3 cross-sectional studies ^#^	2 (low)
	**Factors decreasing confidence**	Risk of bias	Very serious ^a^	Downgrading
		Inconsistency	Limited ^b^	No downgrading
		Indirectness	None ^c^	No downgrading
		Imprecision	Serious^d^	Downgrading
		Publication bias	NA ^e^	No downgrading
	**Factors increasing confidence**	Strength of association	NA ^f^	No upgrading
		Exposure-response gradient	NA ^f^	No upgrading
		Possible confounding	Serious bias cannot be ruled out ^g^	No upgrading
	**Overall judgement of quality of evidence**			0 (very low)

^#^ Since only cross-sectional studies were available, we started with a grading of “low” (2); ^a^ Methods used to select the population: response rates were in two of the three studies below 60%. The participants were randomly selected, taking into account the distance between their house and a wind turbine (park); diabetes was in all cases measured by means of a questionnaire; ^b^ Results across studies differed in the magnitude and direction of effect estimates (see Figure 7.1 of the complete review); ^c^ The evaluated studies assessed population, exposure, and outcome of interest; ^d^ We considered the results to be imprecise: We assessed that the number of cases is probably lower than 200. The 95% CIs of the separate studies contained values below 0.5 and above 2.0; ^e^ Due to the low number of available effect estimates it was not possible to test for publication bias or small study bias; ^f^ Only one of the evaluated studies found that We decided not to carry out a meta-analysis; ^g^ The studies were unable to adjust for important confounders.

**Table A42:** Summary of findings table for the association between aircraft noise exposure and the incidence of diabetes.

Question	Does Exposure to Aircraft Noise Increase the Risk of Diabetes			
**People**	Adult population (men and women)			
**Setting**	Residential setting: people living in Stockholm (Sweden)			
**Outcome**	The incidence of diabetes			
**Summary of findings**	RR per 10 dB increase in aircraft noise level (L_DEN_)		0.99 (95% CI: 0.47–2.09)	
	Number of participants (^#^ studies)		5156 (1)	
	Number of cases		159	
			**Rating**	**Adjustment to rating**
**Quality assessment**	**Starting rating**		1 cohort ^#^	4 (high)
	**Factors decreasing confidence**	Risk of bias	Limited ^a^	No downgrading
		Inconsistency	NA ^b^	No downgrading
		Indirectness	None ^c^	No downgrading
		Imprecision	Serious ^d^	Downgrading
		Publication bias	NA ^e^	No downgrading
	**Factors increasing confidence**	Strength of association	NA ^f^	No upgrading
		Exposure-response gradient	No evidence of an exposure-response gradient ^f^	No upgrading
		Possible confounding	No conclusions can be drawn ^g^	No upgrading
	**Overall judgement of quality of evidence**			2 (low) ^h^

^#^ Since we have a cohort study, we start at 4 (high evidence; ^a^ The loss-to-follow-up was estimated as > 20%; ^b^ Since only one study is available, this criterion is not applicable; ^c^ The study assessed population, exposure and outcome of interest; ^d^ Although the number of cases was large, the 95% CI was judged as not sufficiently narrow; ^e^ Since the results of only one study were available it was not possible to test for publication bias or small study bias; ^f^ The evaluated study found that the risk of diabetes decreased when air traffic noise level increased (RR per 10 dB < 1). No evidence of an exposure-response gradient was found: the evaluated study found an non-significant effect size of 0.99 per 10 dB; ^g^ We were not able to draw any conclusions whether possible residual confounders or biases would reduce our effect estimate; ^h^ We graded the overall quality of the evidence to be “moderate” (3). Since only one study was available, we downgraded the overall level of evidence to “low” (2).

**Table A43:** Summary of findings table for the association between road traffic noise exposure and the incidence of diabetes.

Question	Does Exposure to Road Traffic Noise Increase the Risk of Diabetes			
**People**	Adult population (men and women)			
**Setting**	Residential setting: people living in cities in Denmark			
**Outcome**	The incidence of diabetes			
**Summary of findings**	RR per 10 dB increase in road traffic noise level (L_DEN_)		1.08 (95% CI: 1.02–1.14)	
	Number of participants (^#^ studies)		57,053 (1)	
	Number of cases		2752	
			**Rating**	**Adjustment to rating**
**Quality assessment**	**Starting rating**		1 cohort ^#^	4 (high)
	**Factors decreasing confidence**	Risk of bias	Limited ^a^	No downgrading
		Inconsistency	NA ^b^	No downgrading
		Indirectness	None ^c^	No downgrading
		Imprecision	Limited ^d^	No downgrading
		Publication bias	NA ^e^	No downgrading
	**Factors increasing confidence**	Strength of association	Small ^f^	No upgrading
		Exposure-response gradient	Evidence of a significant exposure-response gradient ^f^	Upgrading
		Possible confounding	No conclusions can be drawn ^g^	No upgrading
	**Overall judgement of quality of evidence**			3 (moderate) ^h^

^#^ Since one cohort study is available, we started with a grading of “high” (4); ^a^ The quality of the study was judged as high; ^b^ Since only one study is available, this criterion is not applicable; ^c^ The study assessed population, exposure and outcome of interest; ^d^ We considered the results of the study to be precise: The number of cases was large, and the 95% CI was sufficiently narrow; ^e^ Since the results of only one study were available it was not possible to test for publication bias or small study bias; ^f^ The evaluated study found that the risk of diabetes increased when road traffic noise level increased (RR per 10 dB < 1). There was evidence of a significant exposure-response gradient: In the evaluated study a statistically significant RR of 1.08 per 10 dB across the noise range of 50–70 dB was found. This means that if the road traffic noise level increases from 50 to 70 dB, the RR = 1.17; ^g^ We were not able to draw any conclusions whether possible residual confounders or biases would reduce our effect estimate; ^h^ We graded the overall quality of the evidence to be “high” (4). Since only one study was available, we downgraded the overall level of evidence to “moderate” (3).

**Table A44:** Summary of findings table for the association between rail traffic noise exposure and the incidence of diabetes.

Question	Does Exposure to Rail Traffic Noise Increase the Risk of Diabetes			
**People**	Adult population (men and women)			
**Setting**	Residential setting: people living in cities in Denmark			
**Outcome**	The incidence of diabetes			
**Summary of findings**	RR per 10 dB increase in aircraft noise level (L_DEN_)		0.97 (95% CI: 0.89–1.05)	
	Number of participants (^#^ studies)		57,053 (1)	
	Number of cases		2752	
			**Rating**	**Adjustment to rating**
**Quality assessment**	**Starting rating**		1 cohort ^#^	4 (high)
	**Factors decreasing confidence**	Risk of bias	Limited ^a^	No downgrading
		Inconsistency	NA ^b^	No downgrading
		Indirectness	None ^c^	No downgrading
		Imprecision	Limited ^d^	No downgrading
		Publication bias	NA ^e^	No downgrading
	**Factors increasing confidence**	Strength of association	NA ^f^	No upgrading
		Exposure-response gradient	No evidence of an exposure-response gradient ^f^	No upgrading
		Possible confounding	No conclusions can be drawn ^g^	No upgrading
	**Overall judgement of quality of evidence**			3 (moderate) ^h^

^#^ Since, a cohort study is available, we started with a grading of “high” (4); ^a^ The quality of the study was judged as high; ^b^ Since only one study is available, this criterion is not applicable; ^c^ The study assessed population, exposure and outcome of interest; ^d^ We considered the results of the studies as precise: the number of cases was large, and the 95% CI was judged as sufficiently narrow; ^e^ Since the results of only one study were available it was not possible to test for publication bias or small study bias; ^f^ The evaluated study found that the risk of diabetes decreased when rail traffic noise level increased (RR per 10 dB < 1). No evidence of an exposure-response gradient was found: the evaluated study found a non-significant effect size of 0.97 per 10 dB; ^g^ We were not able to draw any conclusions whether possible residual confounders or biases would reduce our effect estimate; ^h^ We graded the overall quality of the evidence to be “high” (4). Since only one study was available, we downgraded the overall level of evidence to “moderate” (3).

## Appendix G. Summary of Findings Tables Dealing with Studies on the Impact of Noise on Obesity

This appendix presents the summary of findings tables dealing with the studies on the impact of noise on obesity. An extensive description and the reasoning behind these tables can be found in the complete review in Section 11.5.

**Table A45:** Summary of findings table for the association between aircraft noise exposure and the change in Body Mass Index.

Question	Does Exposure to Aircraft Noise Increase the Risk of Obesity			
**People**	Adult population (men and women)			
**Setting**	Residential setting: people living in Stockholm in areas located around the airport			
**Outcome**	Change in BMI (kg/m^3^)			
**Summary of findings**	Change in BMI per 10 dB increase in aircraft noise level (L_DEN_)		0.14 (95% CI: −0.18–0.45) kg/m^2^	
	Number of participants (^#^ studies)		5156 (1)	
	Number of cases		NR	
			**Rating**	**Adjustment to rating**
**Quality assessment**	**Starting rating**		1 cohort study ^#^	4 (high)
	**Factors decreasing confidence**	Risk of bias	Limited ^a^	No downgrading
		Inconsistency	NA ^b^	No downgrading
		Indirectness	None ^c^	No downgrading
		Imprecision	Serious ^d^	Downgrading
		Publication bias	NA ^e^	No downgrading
	**Factors increasing confidence**	Strength of association	Small ^f^	No upgrading
		Exposure-response gradient	Evidence of a non- significant exposure-response gradient ^f^	No upgrading
		Possible confounding	No conclusions can be drawn ^g^	No upgrading
	**Overall judgement of quality of evidence**			2 (low) ^#^

^#^ Since a cohort study was available, we started with a grading of “high” (4); ^a^ The quality of the study was judged as high; ^b^ Since only one study is available, this criterion is not applicable; ^c^ The study assessed population, exposure and outcome of interest; ^d^ We considered the results to be imprecise: The standard deviation of the reported effect size was larger than the mean difference in BMI; ^e^ Since the results of only one study were available, it was not possible to test for publication bias or small study bias; ^f^ In the evaluated study, a harmful effect of noise was found. There was evidence of a non-significant exposure-response gradient: we found a non-significant effect size of 0.14 kg/m^2^ per 10 dB. The noise range of the study under evaluation was 48–65 dB. This means that in case the air traffic noise level increases from 48 to 65 dB, the BMI increased with 0.24 kg/m^2^ (this is less than 3–5% change in BMI, which is considered clinically significant); ^g^ We were not able to draw any conclusions whether possible residual confounders or biases would reduce our effect estimate; ^h^ We graded the overall quality of the evidence to be “moderate” (3). Because only one study was available, we downgraded the overall quality of evidence to “low” (2).

**Table A46:** Summary of findings table for the association between road traffic noise exposure and the change in Body Mass Index.

Question	Does Exposure to Road Traffic Noise Increase the Risk of Obesity			
**People**	Adult population (men and women)			
**Setting**	Residential setting: people living in Stockholm in areas located around the airport (Sweden), people living in Oslo (Norway), People living in Aarhus or Copenhagen (Denmark)			
**Outcome**	Change in BMI (kg/m^3^)			
**Summary of findings**	Change in BMI per 10 dB increase in road traffic noise level (L_DEN_)		0.03 (95% CI: −0.10–0.15) kg/m^2^	
	Number of participants (^#^ studies)		71,431 (3)	
	Number of cases		NR	
			**Rating**	**Adjustment to rating**
**Quality assessment**	**Starting rating**		3 cross-sectional studies ^#^	2 (low)
	**Factors decreasing confidence**	Risk of bias	Limited ^a^	No downgrading
		Inconsistency	Serious ^b^	Downgrading
		Indirectness	None ^c^	No downgrading
		Imprecision	Serious ^d^	Downgrading
		Publication bias	NA ^e^	No downgrading
	**Factors increasing confidence**	Strength of association	Small ^f^	No upgrading
		Exposure-response gradient	Evidence of a non- significant exposure-response gradient ^f^	No upgrading
		Possible confounding	No conclusions can be drawn ^g^	No upgrading
	**Overall judgement of quality of evidence**			0 (very low)

^#^ Since only cross-sectional studies were available, we started with a grading of “low” (2); ^a^ The quality of the studies was judged as high; ^b^ Results across studies differed in the magnitude and direction of effect estimate (see Figure 8.1 of the complete review). This was confirmed by the results of the heterogeneity analysis, demonstrating “strong” heterogeneity (I^2^_residual_ = 84.4%); ^c^ The study assessed population, exposure and outcome of interest. ^d^ We considered the results to be imprecise: The standard deviation of the reported effect size was larger than the mean difference in BMI; ^e^ Since the number of available estimates was small, it was not possible to test for publication bias or small study bias; ^f^ In one of the evaluated studies, a harmful effect of noise was found. There was evidence of a non-significant exposure-response gradient: After aggregating the results of the studies, we found a non-significant effect size of 0.03 kg/m^2^ per 10 dB. The noise range of the studies under evaluation was ~40–65 dB. This means that if the road traffic noise level increases from 40 to 65 dB, the BMI increased with 0.08 kg/m^2^ (this is probably less than 3–5% change in BMI, which is considered clinically significant); ^g^ We were not able to draw any conclusions whether possible residual confounders or biases would reduce our effect estimate.

**Table A47:** Summary of findings table for the association between rail traffic noise exposure and the change in Body Mass Index.

Question	Does Exposure to Rail Traffic Noise Increase the Risk of Obesity			
**People**	Adult population (men and women)			
**Setting**	Residential setting: people living in Stockholm in areas located around the airport (Sweden), and people living in Aarhus or Copenhagen (Denmark)			
**Outcome**	Change in BMI (kg/m^3^)			
**Summary of findings**	Change in BMI per 10 dB increase in rail traffic noise level (L_DEN_)		-	
	Number of participants (^#^ studies)		57,531 (2)	
	Number of cases		NR	
			**Rating**	**Adjustment to rating**
**Quality assessment**	**Starting rating**		2 cross-sectional studies ^#^	2 (low)
	**Factors decreasing confidence**	Risk of bias	Limited ^a^	No downgrading
		Inconsistency	Serious ^b^	Downgrading
		Indirectness	None ^c^	No downgrading
		Imprecision	Limited ^d^	No downgrading
		Publication bias	NA ^e^	No downgrading
	**Factors increasing confidence**	Strength of association	NA ^f^	No upgrading
		Exposure-response gradient	NA ^f^	No upgrading
		Possible confounding	No conclusions can be drawn ^g^	No upgrading
	**Overall judgement of quality of evidence**			1 (very low)

^#^ Since only cross-sectional studies were available, we started with a grading of “low” (2); ^a^ The quality of the studies was judged as high; ^b^ Results varied between the studies; ^c^ Results across studies differed in the magnitude of effect estimates. The direction of the effects was consistent; ^c^ The study assessed population, exposure and outcome of interest; ^d^ We considered the results to be precise: For both studies, the standard deviations of the reported effect were smaller than the reported effect size; ^e^ Since the number of available estimates was small, it was not possible to test for publication bias or small study bias; ^f^ Both studies found a harmful effect of rail traffic noise. We decided not to carry out a meta-analysis; ^g^ Residual confounding primarily due to the way exposure was assessed, cannot be ruled out. For the other factors, we were not able to draw any conclusions whether possible residual confounders or biases would reduce our effect estimate.

**Table A48:** Summary of findings table for the association between aircraft noise exposure and the change in waist circumference.

Question	Does Exposure to Aircraft Noise Increase the Risk of Obesity			
**People**	Adult population (men and women)			
**Setting**	Residential setting: people living in Stockholm in areas located around the airport			
**Outcome**	Change in waist circumference (cm)			
**Summary of findings**	Change in waist circumference per 10 dB increase in aircraft noise level (L_DEN_)		3.46 (95% CI: 2.13–4.77) cm	
	Number of participants (^#^ studies)		5156 (1)	
	Number of cases		NR	
			**Rating**	**Adjustment to rating**
**Quality assessment**	**Starting rating**		1 cohort study ^#^	4 (high)
	**Factors decreasing confidence**	Risk of bias	Limited ^a^	No downgrading
		Inconsistency	NA ^b^	No downgrading
		Indirectness	None ^c^	No downgrading
		Imprecision	Limited ^d^	No downgrading
		Publication bias	NA ^e^	No downgrading
	**Factors increasing confidence**	Strength of association	Large ^f^	Upgrading
		Exposure-response gradient	Evidence of a significant exposure-response gradient ^f^	Upgrading
		Possible confounding	No conclusions can be drawn ^g^	No upgrading
	**Overall judgement of quality of evidence**			3 (moderate) ^h^

^#^ Since a cohort study was available, we started with a grading of “high” (4); ^a^ The quality of the study was judged as high; ^b^ Since only one study is available, this criterion is not applicable; ^c^ The study assessed population, exposure and outcome of interest; ^d^ We considered the results of the study to be precise: The standard deviation of the reported effect size was smaller than the mean difference in waist circumference; ^e^ Since the results of only one study were available, it was not possible to test for publication bias or small study bias; ^f^ The study found a harmful effect of aircraft noise. There was evidence of a significant exposure-response gradient: we found a significant effect size of 3.46 cm per 10 dB. The noise range of the study under evaluation was 48–65 dB. This means that if the air traffic noise level increases from 48 to 65 dB, the waist circumference increased more than 5.88 cm; ^g^ We were not able to draw any conclusions whether possible residual confounders or biases would reduce our effect estimate; ^h^ We graded the overall quality of the evidence to be ”high” (4). Because only one study was available, we downgraded the overall quality of evidence to “moderate” (3).

**Table A49:** Summary of findings table for the association between road traffic noise exposure and the change in waist circumference.

Question	Does Exposure to Road Traffic Noise Increase the Risk of Obesity			
**People**	Adult population (men and women)			
**Setting**	Residential setting: people living in Stockholm in areas located around the airport (Sweden), people living in Oslo (Norway), People living in Aarhus or Copenhagen (Denmark)			
**Outcome**	Change in waist circumference (cm)			
**Summary of findings**	Change in waist circumference per 10 dB increase in road traffic noise level (L_DEN_)		0.17 (95% CI: −0.06–0.40) cm	
	Number of participants (^#^ studies)		71,431 (3)	
	Number of cases		NR	
			**Rating**	**Adjustment to rating**
**Quality assessment**	**Starting rating**		3 cross-sectional studies ^#^	2 (low)
	**Factors decreasing confidence**	Risk of bias	Limited ^a^	No downgrading
		Inconsistency	Serious ^b^	Downgrading
		Indirectness	None ^c^	No downgrading
		Imprecision	Serious ^d^	No downgrading
		Publication bias	NA ^e^	No downgrading
	**Factors increasing confidence**	Strength of association	Small ^f^	No upgrading
		Exposure-response gradient	Evidence of a non- significant exposure-response gradient ^f^	No upgrading
		Possible confounding	No conclusions can be drawn ^g^	No upgrading
	**Overall judgement of quality of evidence**			1 (very low)

^#^ Since only cross-sectional studies were available, we started with a grading of “low” (2); ^a^ The quality of the studies was judged as high; ^b^ Results across studies differed in the magnitude and direction of effect estimate (see Figure 8.1 of the complete review). This was confirmed by the results of the heterogeneity analysis, demonstrating “strong” heterogeneity (I^2^_residual_ = 84.4%); ^c^ The study assessed population, exposure and outcome of interest; ^d^ We considered the results to be precise enough: The standard deviation of the reported effect size was smaller than the mean difference in waist circumference; ^e^ Since the number of available estimates was small, it was not possible to test for publication bias or small study bias; ^f^ Two studies found a harmful effect of road traffic noise. There was evidence of a non- significant exposure-response gradient: After aggregating the results of the three evaluated studies, we found a non-significant effect size of 0.17 per 10 dB. The noise range of the study under evaluation was ~40–65 dB. This means that if the road traffic noise level increases from 40 to 65 dB, the waist circumference increased with 0.43 cm (this is probably less than 3-5% change in waist circumference, which is considered clinically significant); ^g^ Residual confounding primarily due to the way exposure was assessed cannot be ruled out. For the rest we were not able to draw any conclusions whether possible residual confounders or biases would reduce our effect estimate.

**Table A50:** Summary of findings table for the association between rail traffic noise exposure and the change in waist circumference.

Question	Does Exposure to Rail Traffic Noise Increases the Risk of Obesity			
**People**	Adult population (men and women)			
**Setting**	Residential setting: people living in Stockholm in areas located around the airport (Sweden), and people living in Aarhus or Copenhagen (Denmark)			
**Outcome**	Change in waist circumference (cm)			
**Summary of findings**	Change in waist circumference per 10 dB increase in rail traffic noise level (L_DEN_)		-	
	Number of participants (^#^ studies)		57,531 (2)	
	Number of cases		NR	
			**Rating**	**Adjustment to rating**
**Quality assessment**	**Starting rating**		2 cross-sectional studies ^#^	2 (low)
	**Factors decreasing confidence**	Risk of bias	Limited ^a^	No downgrading
		Inconsistency	Limited ^b^	No downgrading
		Indirectness	None ^c^	No downgrading
		Imprecision	Limited ^d^	No downgrading
		Publication bias	NA ^e^	No downgrading
	**Factors increasing confidence**	Strength of association	NA ^f^	No upgrading
		Exposure-response gradient	NA ^f^	No upgrading
		Possible confounding	No conclusions can be drawn ^g^	No upgrading
	**Overall judgement of quality of evidence**			2 (low)

^#^ Since only cross-sectional studies were available, we started with a grading of “low” (2); ^a^ The quality of the studies was judged as high; ^b^ Results across studies only differed in magnitude of effect estimates; ^c^ The study assessed population, exposure and outcome of interest; ^d^ We considered the results to be precise: For both studies, the standard deviations of the reported effect were smaller than the reported effect size; ^e^ Since the number of available estimates was small, it was not possible to test for publication bias or small study bias; ^f^ Both studies found a harmful effect of rail traffic noise. We decided not to carry out a meta-analysis; ^g^ We were not able to draw any conclusions whether possible residual confounders or biases would reduce our effect estimate.

## Appendix H. Summary of Findings Tables Dealing with Studies on the Impact of Noise on Children’s Blood Pressure

This appendix presents the summary of findings tables dealing with the studies on the impact of noise on children’s blood pressure. An extensive description and the reasoning behind these tables can be found in the complete review in Section 11.6.

**Table A51:** Summary of findings table for the association between aircraft noise exposure at home and the change in systolic blood pressure in children.

Question	Does Exposure to Aircraft Noise Affect Blood Pressure			
**People**	Children (boys and girls)			
**Setting**	Residential setting: Children (aged 6–11 years) living in cities around Schiphol Amsterdam airport (The Netherlands), London Heathrow (United Kingdom) and Kingsford-Smith airport (Australia)			
**Outcome**	Change in systolic blood pressure (mmHg)			
**Summary of findings**	Change in systolic blood pressure level per 10 dB increase in aircraft noise level (L_DEN_)		-	
	Number of participants (^#^ studies)		2013 (2)	
	Number of cases		NR	
			**Rating**	**Adjustment to rating**
**Quality assessment**	**Starting rating**		2 cross-sectional studies ^#^	2 (low)
	**Factors decreasing confidence**	Risk of bias	A lot is unclear ^a^	Downgrading
		Inconsistency	Serious ^b^	Downgrading
		Indirectness	None ^c^	No downgrading
		Imprecision	Serious ^d^	Downgrading
		Publication bias	NA ^e^	No downgrading
	**Factors increasing confidence**	Strength of association	NA ^f^	No upgrading
		Exposure-response gradient	NA ^f^	No upgrading
		Possible confounding	No conclusions can be drawn ^g^	No upgrading
	**Overall judgement of quality of evidence**			0 (very low)

^#^ Since only cross-sectional studies were available, we started with a grading of “low” (2); ^a^ The quality of the studies was judged as low, since response rates in both studies were higher than 60%, and because of the difficulty to judge the quality of the blood pressure measurements; ^b^ One study found a positive effect; the other found a negative effect (see Figure 9.1 of the complete review); ^c^ The studies assessed population, exposure and outcome of interest; ^d^ We considered the results to be imprecise: The standard deviation of the reported effect size was larger than the mean difference in blood pressure; ^e^ Since the results of only two studies were available it was not possible to test for publication bias or small study bias; ^f^ One of the studies found a harmful effect of noise. It was not possible to combine the results of both studies. A meta-analysis was not carried out; ^g^ We were not able to draw any conclusions whether possible residual confounders or biases would reduce our effect estimate.

**Table A52:** Summary of findings table for the association between aircraft noise exposure at home and the change in diastolic blood pressure in children.

Question	Does Exposure to Aircraft Noise Affect Blood Pressure			
**People**	Children (boys and girls)			
**Setting**	Residential setting: Children (aged 6–11 years) living in cities around Schiphol Amsterdam airport (The Netherlands), London Heathrow (United Kingdom) and Kingsford-Smith airport (Australia)			
**Outcome**	Change in diastolic blood pressure (mmHg)			
**Summary of findings**	Change in diastolic blood pressure level per 10 dB increase in aircraft noise level (L_DEN_)		-	
	Number of participants (^#^ studies)		2013 (2)	
	Number of cases		NR	
			**Rating**	**Adjustment to rating**
**Quality assessment**	**Starting rating**		2 cross-sectional studies ^#^	2 (low)
	Factors decreasing confidence	Risk of bias	A lot is unclear ^a^	Downgrading
		Inconsistency	Serious ^b^	Downgrading
		Indirectness	None ^c^	No downgrading
		Imprecision	Serious ^d^	Downgrading
		Publication bias	NA ^e^	No downgrading
	Factors increasing confidence	Strength of association	NA ^f^	No upgrading
		Exposure-response gradient	NA ^f^	No upgrading
		Possible confounding	No conclusions can be drawn ^g^	No upgrading
	**Overall judgement of quality of evidence**			0 (very low)

^#^ Since only cross-sectional studies were available, we started with a grading of “low” (2); ^a^ The quality of the studies was judged as low, since response rates in both studies were higher than 60% and because of the difficulty to judge the quality of the blood pressure measurements; ^b^ One study found a positive effect; the other found a negative effect (see Figure 9.2 of the complete review); ^c^ The studies assessed population, exposure and outcome of interest; ^d^ We considered the results to be imprecise: The standard deviation of the reported effect size was larger than the mean difference in blood pressure; ^e^ Since the results of only two studies were available it was not possible to test for publication bias or small study bias; ^f^ One of the evaluated studies found a harmful effect of noise. It was not possible to combine the results of both studies. A meta-analysis was not carried out; ^g^ We were not able to draw any conclusions whether possible residual confounders or biases would reduce our effect estimate.

**Table A53:** Summary of findings table for the association between aircraft noise exposure at school and the change in systolic blood pressure in children.

Question	Does Exposure to Aircraft Noise Affect Blood Pressure			
**People**	Children (boys and girls)			
**Setting**	Educational setting: Children (aged 6–11 years) visiting primary schools in cities around Schiphol Amsterdam airport (The Netherlands), London Heathrow (United Kingdom) and Kingsford-Smith airport (Australia)			
**Outcome**	Change in systolic blood pressure (mmHg)			
**Summary of findings**	Change in systolic blood pressure level per 10 dB increase in aircraft noise level (L_DEN_)		-	
	Number of participants (^#^ studies)		2013 (2)	
	Number of cases		NR	
			**Rating**	**Adjustment to rating**
**Quality assessment**	**Starting rating**		2 cross-sectional studies ^#^	2 (low)
	**Factors decreasing confidence**	Risk of bias	A lot is unclear ^a^	Downgrading
		Inconsistency	Serious ^b^	Downgrading
		Indirectness	None ^c^	No downgrading
		Imprecision	Serious ^d^	Downgrading
		Publication bias	NA ^e^	No downgrading
	**Factors increasing confidence**	Strength of association	NA ^f^	No upgrading
		Exposure-response gradient	NA ^f^	No upgrading
		Possible confounding	No conclusions can be drawn ^g^	No upgrading
	**Overall judgement of quality of evidence**			0 (very low)

^#^ Since only cross-sectional studies were available, we started the grading with “low” (2); ^a^ The quality of the studies was judged as low, since response rates in both studies were higher than 60% and because of the difficulty to judge the quality of the blood pressure measurements; ^b^ One study found a positive effect; the other found a negative effect (see Figure 9.1 of the complete review); ^c^ The studies assessed population, exposure and outcome of interest; ^d^ The standard deviation of the reported effect size was larger than the mean difference in blood pressure; ^e^ Since the results of only two studies were available it was not possible to test for publication bias or small study bias; ^f^ It was not possible to combine the results of both studies. A meta-analysis was not carried out; ^g^ We were not able to draw any conclusions whether possible residual confounders or biases would reduce our effect estimate.

**Table A54:** Summary of findings table for the association between aircraft noise exposure at school and the change in diastolic blood pressure in children.

Question	Does Exposure to Aircraft Noise Affect Blood Pressure			
**People**	Children (boys and girls)			
**Setting**	Educational setting: Children (aged 6–11 years) visiting primary schools in cities around Schiphol Amsterdam airport (The Netherlands), London Heathrow (United Kingdom) and Kingsford-Smith airport (Australia)			
**Outcome**	Change in diastolic blood pressure (mmHg)			
**Summary of findings**	Change in diastolic blood pressure level per 10 dB increase in aircraft noise level (L_DEN_)		-	
	Number of participants (^#^ studies)		2013 (2)	
	Number of cases		NR	
			**Rating**	**Adjustment to rating**
**Quality assessment**	**Starting rating**		2 cross-sectional studies ^#^	2 (low)
	**Factors decreasing confidence**	Risk of bias	A lot is unclear ^a^	Downgrading
		Inconsistency	Serious ^b^	Downgrading
		Indirectness	None ^c^	No downgrading
		Imprecision	Serious ^d^	Downgrading
		Publication bias	NA ^e^	No downgrading
	**Factors increasing confidence**	Strength of association	NA ^f^	No upgrading
		Exposure-response gradient	NA ^f^	No upgrading
		Possible confounding	No conclusions can be drawn ^g^	No upgrading
	**Overall judgement of quality of evidence**			0 (very low)

^#^ Since only cross-sectional studies were available, we started with a grading of ”low” (2); ^a^ The quality of the studies was judged as low, since response rates in both studies were higher than 60% and because of the difficulty to judge the quality of the blood pressure measurements; ^b^ One study found a positive effect; the other found a negative effect (see Figure 9.2 of the complete review); ^c^ The studies assessed population, exposure and outcome of interest; ^d^ The standard deviation of the reported effect size was larger than the mean difference in blood pressure; ^e^ Since the results of only two studies were available it was not possible to test for publication bias or small study bias; ^f^ It was not possible to combine the results of both studies. A meta-analysis was not carried out; ^g^ We were not able to draw any conclusions whether possible residual confounders or biases would reduce our effect estimate.

**Table A55:** Summary of findings table for the association between road traffic noise exposure at home and the change in systolic blood pressure in children.

Question	Does Exposure to Road Traffic Noise Affect Blood Pressure			
**People**	Children (boys and girls)			
**Setting**	Residential setting: Children (aged 6–11 years) living in cities in The Netherlands, the United Kingdom, Germany, Croatia, Serbia and the United States of America			
**Outcome**	Change in systolic blood pressure (mmHg)			
**Summary of findings**	Change in systolic blood pressure level per 10 dB increase in road traffic noise level (L_DEN_)		0.08 (95% CI: −0.48–0.64) mmHg	
	Number of participants (^#^ studies)		4197 (6)	
	Number of cases		NR	
			**Rating**	**Adjustment to rating**
**Quality assessment**	**Starting rating**		6 cross-sectional studies ^#^	2 (low)
	**Factors decreasing confidence**	Risk of bias	Serious ^a^	Downgrading
		Inconsistency	Serious ^b^	Downgrading
		Indirectness	None ^c^	No downgrading
		Imprecision	Serious ^d^	Downgrading
		Publication bias	NA ^e^	No downgrading
	**Factors increasing confidence**	Strength of association	NA ^f^	No upgrading
		Exposure-response gradient	Evidence of a non-significant exposure-response gradient ^f^	No upgrading
		Possible confounding	No conclusions can be drawn ^g^	No upgrading
	**Overall judgement of quality of evidence**			0 (very low)

^#^ Since only cross-sectional studies were available, we started with a grading of “low” (2); ^a^ The quality of the studies was judged as low, since response rates in both studies were higher than 60% and because of the difficulty to judge the quality of the blood pressure measurements. Also studies were not always able to adjust for confounding or were able to attribute individual exposure estimates; ^b^ Results across studies differed in the magnitude and direction of effect estimates (see Figure 9.1 of the complete review). This was not confirmed by the results of the heterogeneity analysis, demonstrating only “low” heterogeneity (I^2^_residual_ = 8.9%); ^c^ The studies assessed population, exposure and outcome of interest; ^d^ We considered the results to be imprecise: The standard deviation of the reported effect size was larger than the mean difference in blood pressure; ^e^ Since the number of available effect estimates was less than 10, it was not possible to test for publication bias or small study bias; ^f^ Three of the evaluated studies found a harmful effect of noise. There was evidence of a non-significant exposure-response gradient: after combining the results of the evaluated studies, we found a non-significant effect size of 0.08 mmHg per 10 dB. The noise range was ~35–80 dB. This means that if the road traffic noise level increases from 35 to 80 dB, the blood pressure increased with 0.36 mmHg; ^g^ We were not able to draw any conclusions whether possible residual confounders or biases would reduce our effect estimate.

**Table A56:** Summary of findings table for the association between road traffic noise exposure at home and the change in diastolic blood pressure in children.

Question	Does Exposure to Road Traffic Noise Affect Blood Pressure			
**People**	Children (boys and girls)			
**Setting**	Residential setting: Children (aged 6–11 years) living in cities in The Netherlands, the United Kingdom, Germany, Croatia, Serbia and the United States of America			
**Outcome**	Change in diastolic blood pressure (mmHg)			
**Summary of findings**	Change in diastolic blood pressure level per 10 dB increase in road traffic noise level (L_DEN_)		0.47 (95% CI: −0.30–1.24) mmHg	
	Number of participants (^#^ studies)		4197 (6)	
	Number of cases		NR	
			**Rating**	**Adjustment to rating**
**Quality assessment**	**Starting rating**		6 cross-sectional studies ^#^	2 (low)
	**Factors decreasing confidence**	Risk of bias	Serious ^a^	Downgrading
		Inconsistency	Serious ^b^	Downgrading
		Indirectness	None ^c^	No downgrading
		Imprecision	Serious ^d^	Downgrading
		Publication bias	NA ^e^	No downgrading
	**Factors increasing confidence**	Strength of association	NA ^f^	No upgrading
		Exposure-response gradient	Evidence of a non-significant exposure-response gradient ^f^	No upgrading
		Possible confounding	No conclusions can be drawn ^g^	No upgrading
	**Overall judgement of quality of evidence**			0 (very low)

^#^ Since only cross-sectional studies were available, we started with a grading of “low” (2); ^a^ The quality of the studies was judged as low, since response rates in both studies were higher than 60%, and because of the difficulty to judge the quality of the blood pressure measurements. Also studies were not always able to adjust for confounding or were able to attribute individual exposure estimates; ^b^ Results across studies differed in the magnitude and direction of effect estimates (see Figure 9.2 of the complete review). This was confirmed by the results of the heterogeneity analysis, demonstrating “strong” heterogeneity (I^2^_residual_ = 76.0%); ^c^ The studies assessed population, exposure and outcome of interest; ^d^ The results were considered to be imprecise: The standard deviation of the reported effect size was larger than the mean difference in blood pressure; ^e^ Since the number of available effect estimates was less than 10, it was not possible to test for publication bias or small study bias; ^f^ Three of the evaluated studies found a harmful effect of noise. There was evidence of a non-significant exposure-response gradient: After combining the results of the evaluated studies we found a non-significant effect size of 0.47 mmHg per 10 dB. The noise range was ~35–80 dB. This means that if the road traffic noise level increases from 35 to 80 dB, the blood pressure increased with 2.1 mmHg; ^g^ We were not able to draw any conclusions whether possible residual confounders or biases would reduce our effect estimate.

**Table A57:** Summary of findings table for the association between road traffic noise exposure at school and the change in systolic blood pressure in children.

Question	Does Exposure to Road Traffic Noise Affects Blood Pressure			
**People**	Children (boys and girls)			
**Setting**	Educational setting: Children (aged 6–11 years) living in cities in The Netherlands, the United Kingdom, Croatia, Serbia and the United States of America			
**Outcome**	Change in systolic blood pressure (mmHg)			
**Summary of findings**	Change in systolic blood pressure level per 10 dB increase in road traffic noise level (L_DEN_)		−0.60 (95% CI: −1.51–0.30) mmHg	
	Number of participants (^#^ studies)		4520 (5)	
	Number of cases		NR	
			**Rating**	**Adjustment to rating**
**Quality assessment**	**Starting rating**		5 cross-sectional studies ^#^	2 (low)
	**Factors decreasing confidence**	Risk of bias	Serious ^a^	Downgrading
		Inconsistency	Serious ^b^	Downgrading
		Indirectness	None ^c^	No downgrading
		Imprecision	Serious ^d^	Downgrading
		Publication bias	NA ^e^	No downgrading
	**Factors increasing confidence**	Strength of association	NA ^f^	No upgrading
		Exposure-response gradient	No evidence of an exposure-response gradient ^f^	No upgrading
		Possible confounding	No conclusions can be drawn ^g^	No upgrading
	**Overall judgement of quality of evidence**			0 (very low)

^#^ Since we only cross-sectional studies were available, we started with a grading of “low” (2); ^a^ The quality of the studies was judged as low, since response rates in both studies were higher than 60% and because of the difficulty to judge the quality of the blood pressure measurements. Also studies were not always able to adjust for confounding or were able to attribute individual exposure estimates; ^b^ Results across studies differed in the magnitude and direction of effect estimates (see Figure 9.1 of the complete review). This was confirmed by the results of the heterogeneity analysis, demonstrating “moderate” heterogeneity (I^2^_residual_ = 61.6%); ^c^ The studies assessed population, exposure and outcome of interest; ^d^ We considered the results to be imprecise: The standard deviation of the reported effect size was larger than the mean difference in blood pressure; ^e^ Since the number of available effect estimates was less than 10, it was not possible to test for publication bias or small study bias; ^f^ Three studies found a harmful effect. There was no evidence of an exposure-response gradient: after combining the results of the evaluated studies, we found a non-significant effect size of −0.60 mmHg per 10 dB; ^g^ We were not able to draw any conclusions whether possible residual confounders or biases would reduce our effect estimate.

**Table A58:** Summary of findings table for the association between road traffic noise exposure at school and the change in diastolic blood pressure in children.

Question	Does Exposure to Road Traffic Noise Affect Blood Pressure			
**People**	Children (boys and girls)			
**Setting**	Educational setting: Children (aged 6–11 years) living in cities in The Netherlands, the United Kingdom, Croatia, Serbia and the United States of America			
**Outcome**	Change in diastolic blood pressure (mmHg)			
**Summary of findings**	Change in diastolic blood pressure level per 10 dB increase in road traffic noise level (L_DEN_)		0.46 (95% CI: −0.60–1.53) mmHg	
	Number of participants (^#^ studies)		4520 (5)	
	Number of cases		NR	
			**Rating**	**Adjustment to rating**
**Quality assessment**	**Starting rating**		5 cross-sectional studies^#^	2 (low)
	**Factors decreasing confidence**	Risk of bias	Serious ^a^	Downgrading
		Inconsistency	Serious ^b^	Downgrading
		Indirectness	None ^c^	No downgrading
		Imprecision	Serious ^d^	Downgrading
		Publication bias	NA ^e^	No downgrading
	**Factors increasing confidence**	Strength of association	NA ^f^	No upgrading
		Exposure-response gradient	Evidence of a statistically non-significant exposure-response gradient ^f^	No upgrading
		Possible confounding	No conclusions can be drawn ^g^	No upgrading
	**Overall judgement of quality of evidence**			0 (very low)

^#^ Since only cross-sectional studies were available, we started with a grading of “low” (2); ^a^ The quality of the studies was judged as low, since response rates in both studies were higher than 60% and because of the difficulty to judge the quality of the blood pressure measurements. Also studies were not always able to adjust for confounding or were able to attribute individual exposure estimates; ^b^ Results across studies differed in the magnitude and direction of effect estimates (see Figure 9.1 of the complete review). This was not confirmed by the results of the heterogeneity analysis, demonstrating “low” heterogeneity (I^2^_residual_ = 16.0%); ^c^ The studies assessed population, exposure and outcome of interest; ^d^ We considered the results to be imprecise: The standard deviation of the reported effect size was larger than the mean difference in blood pressure; ^e^ Since the number of available effect estimates was less than 10, it was not possible to test for publication bias or small study bias; ^f^ There was evidence of a statistically non-significant exposure-response gradient: after combining the results of the evaluated studies, we found a non-significant effect size of 0.46 mmHg per 10 dB. The noise range was ~35–80 dB. This means that if the road traffic noise level increases from 35 to 80 dB, the blood pressure increased with 2.1 mmHg; ^g^ We were not able to draw any conclusions whether possible residual confounders or biases would reduce our effect estimate.
